# Deletion of hepatic FXR leads to more severe MASH development in female mice

**DOI:** 10.1097/HC9.0000000000000693

**Published:** 2025-05-16

**Authors:** Zakiyah R. Henry, Laura Armstrong, Syeda Maliha, Xuelian Jia, Sophie Gao, Rulaiha E. Taylor, Veronia Basaly, Katherine Otersen, Zhenning Yang, Daniel Rizzolo, Mary Stofan, Vik Meadows, Anisha Bhattacharya, Peihong Zhou, Ill Yang, Anita Brinker, Lanjing Zhou, Hao Zhu, Laurie B. Joseph, Brian Buckley, Tracy Anthony, Lauren Aleksunes, Bo Kong, Grace L. Guo

**Affiliations:** 1 Department of Pharmacology and Toxicology, Rutgers University, Piscataway, New Jersey, USA; 2 Environmental and Occupational Health Science Institute, Rutgers University, Piscataway, New Jersey, USA; 3 Ernest Mario School of Pharmacy, Rutgers University, Piscataway, New Jersey, USA; 4 Rutgers Center for Lipid Research, Rutgers University, New Brunswick, New Jersey, USA; 5 Department of Chemistry and Biochemistry, Rowan University, Glassboro, New Jersey, USA; 6 Division of Biomedical Informatics and Genomics, Tulane University, New Orleans, Louisiana, USA; 7 Department of Veterans Affairs New Jersey Health Care System, East Orange, New Jersey, USA

**Keywords:** bile acid, intestine, liver, steatohepatitis, tissue-specific

## Abstract

**Background:**

The farnesoid X receptor (FXR) has been identified as a therapeutic target for metabolic dysfunction–associated steatohepatitis (MASH). FXR is the major homeostatic regulator of bile acids (BAs) with dysregulation of BAs and/or FXR implicated in the pathogenesis of MASH. Synthetic whole-body FXR agonists have been developed to treat MASH. Although beneficial for MASH treatment, these whole-body modulators contribute to unfavorable side effects such as pruritus and an elevation in low-density liporoteins, thereby highlighting the importance of tissue and cell-restricted modulation of FXR in the development of novel therapeutics for MASH to negate potential harmful off-target effects.

**Methods:**

The objective of this study was to determine the tissue-specific role of FXR in MASH development using male and female wild-type (WT), liver FXR KO (FXR^hep−/−^), intestinal FXR KO (FXR^int−/−^), and whole body FXR KO (FXR KO) mice fed either a low-fat control diet (CTL) or a MASH “Fast Food” (FF) diet.

**Results:**

The results showed, in females, hepatic, but not intestinal, deficiency of FXR was associated with severe liver injury, through increased ALT, ALP, and genes indicative of inflammation and fibrosis when comparing FXR^hep−/−^ versus FXR^int−/−^. Regardless of sex, hepatic FXR deficiency triggered the activation of neuroinflammation and neurodegenerative canonical pathways.

**Conclusions:**

These data suggest that hepatic FXR is more critical in suppressing liver injury during MASH development in female mice. However, this same trend was not clear in the male cohorts, highlighting sex differences and potential roles for sexual dimorphism in MASH development.

## Graphical abstract

**Figure:**
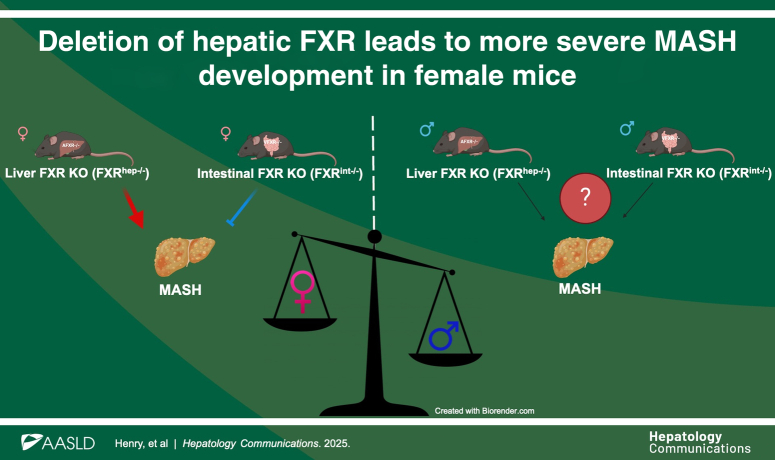

## INTRODUCTION

Approximately 30% of the US population suffers from metabolic dysfunction–associated steatotic liver disease (MASLD), formerly known as NAFLD.^1^ Nearly 25% of the MASLD population will transition to metabolic dysfunction–associated steatohepatitis (MASH). MASH is the more severe and progressive form of fatty liver disease and is characterized by liver steatosis, hepatocellular ballooning, inflammation, and varying degrees of fibrosis.^2^^,^^3^ If fibrosis is not mitigated, end-stage liver disease will manifest in the form of cirrhosis, which increases the risk for HCC or liver transplantation.^4^^,^^5^ MASH is a cause for concern, as MASH-induced cirrhosis is the leading indicator for liver transplantation in many developed countries, including the United States, and its prevalence is rapidly rising.^6^ Furthermore, other than lifestyle changes, treatments are limited, as Resmetirom, a thyroid hormone β partial agonist, is the only FDA-approved therapeutic.^7^ Although there are limited pharmacological agents, there are numerous promising targets including the farnesoid X receptor (FXR).^8^^,^^9^

FXR is a type II nuclear receptor and ligand-activated transcription factor predominantly expressed in the liver and ileum, but also expressed in other tissues such as the lungs, kidneys, adrenal glands, and immune cells.^10^^,^^11^ FXR is known as the master regulator of bile acid (BA) homeostasis, and BAs are endogenous ligands of FXR.^12^ The synthesis of BAs is regulated by FXR via negative feedback mechanisms involving the small heterodimer partner (SHP) in the liver and the fibroblast growth factor 15/19 (*FGF15/19*) in the intestine (ileum) to suppress the gene expression of key rate-limiting enzymes involved in BA synthesis, cholesterol-7α-hydroxylase (CYP7A1) and sterol 12α-hydroxylase (CYP8B1).^13^ The dysregulation of BAs has been implicated in MASH development and progression, partially due to their cytotoxic detergent-like properties. If not properly regulated, accumulated BAs can disrupt cellular membranes and invoke cell death.^14^ Thereby, FXR is critical in protecting the liver from BA-induced liver damage.^15^ In addition, FXR is also crucial for maintaining other proper liver functions, especially during steatohepatitis development by decreasing steatosis, inflammation, and fibrosis.^16^^,^^17^ Previous work from our lab provides the first piece of evidence that FXR deficiency in male mice leads to more severe MASH development.^15^ It is also known that in the clinic, MASH patients have reduced FXR expression and activity compared to non-MASH patients, thereby confirming the importance of FXR in this chronic liver disease.^18^^,^^19^

Because FXR negatively regulates BA production and is deficient in MASH patients, global synthetic ligands of FXR, such as obeticholic acid, cilofexor, and tropifexor, have been developed to treat MASH.^20^^,^^21^ Although beneficial to treat MASH,^22^^–^^24^ these whole-body FXR agonists result in off-target effects such as an imbalance in cholesterol homeostasis by elevating LDL, pruritus, abdominal discomfort, and fatigue.^25^^–^^28^ For these reasons, it is important to identify and understand the underlying mechanisms contributing to the tissue-restricted functionality of FXR in MASH development for the creation of novel and targeted drugs that preserve therapeutic benefits while limiting adverse effects. Thereby, we here examine FXR functionality in a tissue-specific manner between the liver and intestine (ileum) as it relates to MASH development and progression.

## METHODS

### Animals and treatments

Six-week to eight-week old male and female mice in 6 genotypes: wild type (WT, C57BL/6J, *Fxr* ^+/+^), whole-body FXR KO (*Fxr* ^−/−^, FXR KO), liver FXR KO (*Fxr* ^floxed/floxed^, albumin cre (+), FXR^hep−/−^), intestinal FXR KO (*Fxr* ^floxed/floxed^, villin cre (+), FXR^int−/−^), along with the relative floxed/floxed cre (−) controls, were fed either a low-fat control diet (CTL) or a MASH “Fast Food” (FF) diet (Western diet with 21% milk fat, 1.25% cholesterol, and 34% sucrose) for 16 weeks (n=4–20). Body weights were recorded weekly, and an oral glucose tolerance test (GTT) was performed at 12 weeks of feeding.^29^ By the end of the experiment, mice were euthanized. Blood, liver, and small intestine (SI) were collected and frozen in liquid nitrogen. All mice were group-housed and maintained under standard 12-hour light/dark cycles. Food and water were provided *ad libitum* unless otherwise noted. The experiments performed in this study were approved by the Rutgers Institutional Animal Care and Use Committee.

### Serum biochemistry

Serum samples were analyzed for activities of ALT and ALP, along with concentrations of total cholesterol, triglyceride, albumin, and bilirubin using the Heska Element DC5X Blood Chemistry Analyzer (Heska).

### Gene expression

Total RNA was extracted from snap-frozen ileum and liver samples using TRIzol reagent (Thermo Fisher Scientific). Extracted RNA was converted to complementary DNA using reverse transcription. Relative gene expression was determined by real-time quantitative PCR using SYBR green chemistry. Samples were loaded in a 384-well plate and inserted into the Viia7 real-time PCR machine (Life Technologies). All Ct values were converted to delta delta Ct values and normalized to the mRNA levels of a housekeeping gene, *Rps29*. Sequences of primers used can be found in Supplemental Table S1, http://links.lww.com/HC9/B971.

### Liver lipid measurement

Cholesterol and triglycerides were extracted from the liver^30^ and measured using commercially available lipid assay kits (Pointe Scientific) and spectrophotometry. Calculated lipid levels were normalized to the amount of liver tissue used, and data were reported as milligram of lipid per gram of liver weight.

### Histopathology

Liver sections were cut at 3 μm and stained using hematoxylin and eosin for histological assessment (n=3–6). Immunohistochemistry was conducted on formalin-fixed, paraffin-embedded liver sections using rat F4/80 (F4/80; 1:1000; Bio-Rad) or IgG control (Rat IgG 2b Negative Control; 1:1000; Bio-Rad). Semiquantitative analyses based on the percentages of positive stains were performed on 6 representative images per animal per treatment group, photographed at 10× magnification using the Fiji package of ImageJ. Collagen deposition was measured using the staining fast green Sirius red (FGSR). FGSR was prepared using 0.1% (w/v) Direct Red 80 (Sigma-Aldrich) and 0.1% (w/v) fast green FCF (Thermo Fisher Scientific) in saturated aqueous picric acid (Sigma-Aldrich).

### RNA-seq analysis

Total liver and ileal RNAs from FXR^hep−/−^ and FXR^int−/−^ female and male mice (n=3–4 per group) were extracted from frozen tissue via the TRIzol method (Thermo Fisher Scientific). Samples were sent for sequencing to GENEWIZ Multiomics & Synthesis Solutions from Azenta Life Sciences (Azenta US, Inc.). Paired-end sequencing was conducted using the Illumina Next Generation Sequencing platform with the following sequencing configuration: Illumina, 2×150 bp, ~ 350 million paired-end reads (~105 GB), single index, per lane.

### BA extraction and profiling

Total BAs were extracted and purified from all treatment groups for serum, liver, and SI. Samples were prepped and analyzed as described by Taylor et al.^31^ Calculations of BA indices were performed as described by Alamoudi et al.^32^

### Statistical analysis

Data are presented as mean±SD. GraphPad Prism (version 10; GraphPad Software Inc.) was used for statistical analysis. Differences among individual groups were evaluated by 2-way ANOVA followed by Tukey multiple comparison test. Differences were considered statistically significant at *p*<0.05.

## RESULTS

### Hepatic FXR is critical in maintaining liver health in female mice during MASH development

Weekly body weights showed that, regardless of sex, WT and FXR^int−/−^ mice gained significant weight, whereas FXR KO and FXR^hep−/−^ mice were resistant to weight gain (Figure 1A). No changes were seen in GTT results (Supplemental Figure S1, http://links.lww.com/HC9/B971). Although female FXR^hep−/−^ mice were resistant to weight gain, those fed the FF diet had enlarged livers as indicated in the LW/BW ratio data (Figure 1B). Increased LW/BW ratios were also seen in the male WT FF-fed group, but not in any of the conditional KO models. Liver histology mirrored LW/BW ratio data in that the more severe liver damage in the form of steatosis and immune cell infiltration was found in the female FXR^hep−/−^ mice, regardless of diet, and male WT and FXR^int−/−^ mice both on FF diet (Figure 1C).

FIGURE 1Phenotype characterization at the end of 16 weeks on diets for WT, FXR KO, FXR^hep−/−^, and FXR^int−/−^ Mice. (A) Female (top) and male (bottom) body weight change over the course of treatment. (B) Female (left) and male (right) liver-to-body weight ratios. (C) Female (left) and male (right) H&E-stained liver sections. (D) Female (left) and male (right) serum biochemistry: ALT, ALP, cholesterol, and triglycerides. Data represented as mean±SD (n=4–16). Two-way ANOVA; *p<0.05, **p<0.01, ***p<0.001, and ****p<0.0001. *Represents significant difference between diets within the same genotype; ^#^Represents significant difference within diet and sex among different genotypes. Abbreviations: CTL, control; FF, fast food; H&E, hematoxylin and eosin; WT, wild type.

**Figure:**
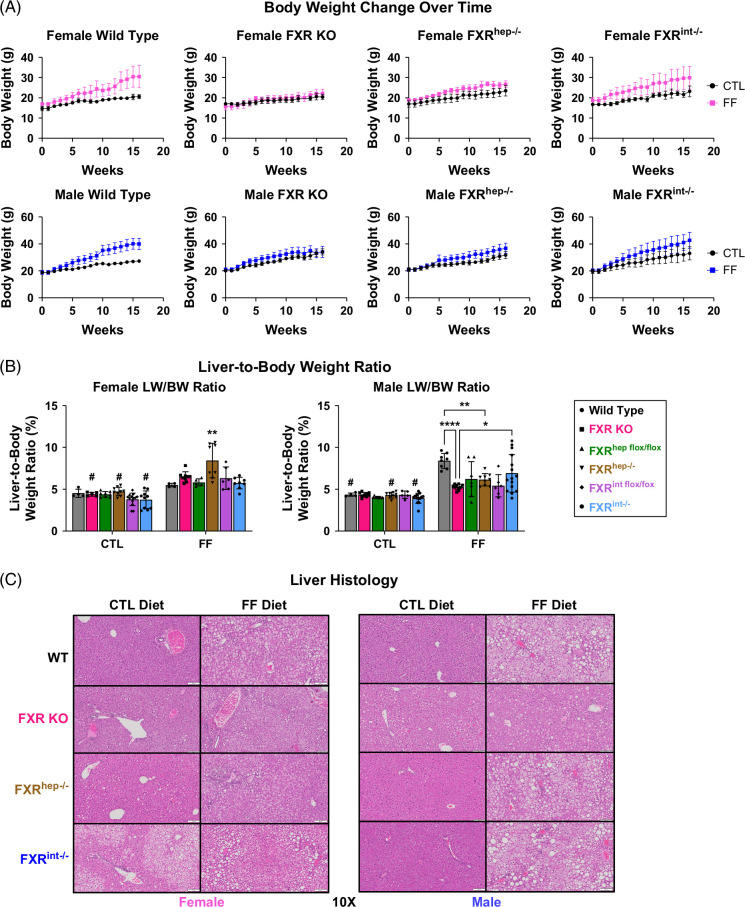

**Figure:**
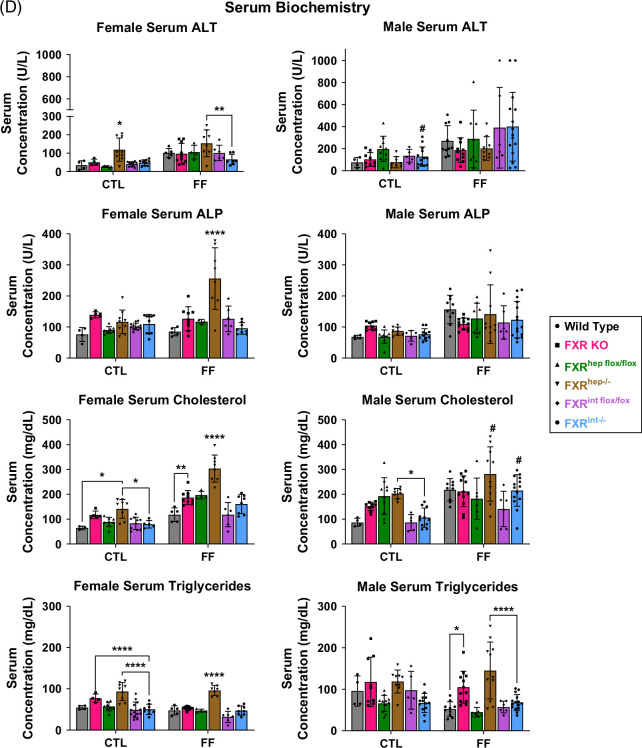

To further characterize model phenotypes, after 16 weeks of feeding, serum biochemistry was analyzed. In the female mice, the FXR^hep−/−^ mice on the FF diet displayed significantly increased levels of ALT, ALP, total cholesterol, and triglycerides compared to FXR KO and FXR^int−/−^ groups (Figure 1D). Furthermore, ALT, total cholesterol, and triglycerides were increased in these same mice on the control diet, resulting in diet-independent and genotype-dependent differences. Male FXR^hep−/−^ mice displayed significantly increased serum lipids, but not liver injury markers, compared to FXR^int−/−^. Female FXR KO and FXR^hep−/−^ mice on FF diet had significantly increased levels of serum BAs compared to FXR^int−/−^. Interestingly, for the males, serum BA levels were significantly increased in WT mice fed the FF diet. There was no difference seen between the tissue-specific FXR KO mice. Serum albumin levels were significantly decreased in male FXR KO mice on FF diet, and there was a trend toward a decrease in female counterparts. No trends were observed in tissue-specific KO mice for serum albumin and total bilirubin (Supplemental Figure S2, http://links.lww.com/HC9/B971).

### Deletion of hepatic FXR triggers upregulation of the classical BA pathway in female mice

To confirm genotypes, qPCR was performed. Fxr was effectively decreased in all tissue-specific FXR KO groups along with the FXR KO groups regardless of sex and diet (Figure 2A). The mRNA levels of direct targets of hepatic and ileal Fxr, Shp, and Fgf15, respectively, were decreased in response to Fxr deficiency, further confirming genotypes.

FIGURE 2FXR signaling. (A) Female (top) and male (bottom) mRNA expression of liver and ileal Fxr and FXR target genes, Shp, and Fgf15, respectively, for WT, FXR KO, FXR^hep−/−^, and FXR^int−/−^. (B) Hepatic (top) and ileal (bottom) BA transporters and binding protein mRNA expression: Abcb11, Slc10a1, Slc10a2, and Fabp6, respectively. (C) Female (left) and male (right) classical (top) and alternative (bottom) mRNA expression of genes in BA synthesis pathways: Cyp7a1, Cyp8b1, Cyp27a1, and Cyp7b1. Data represented as mean±SD (n=4–20). Two-way ANOVA; *p<0.05, **p<0.01, ***p<0.001, and ****p<0.0001. * Represents significant difference between genotypes and within diet; ^#^Represents significant difference within genotype and across diet. Abbreviations: CTL, control; FF, fast food.

**Figure:**
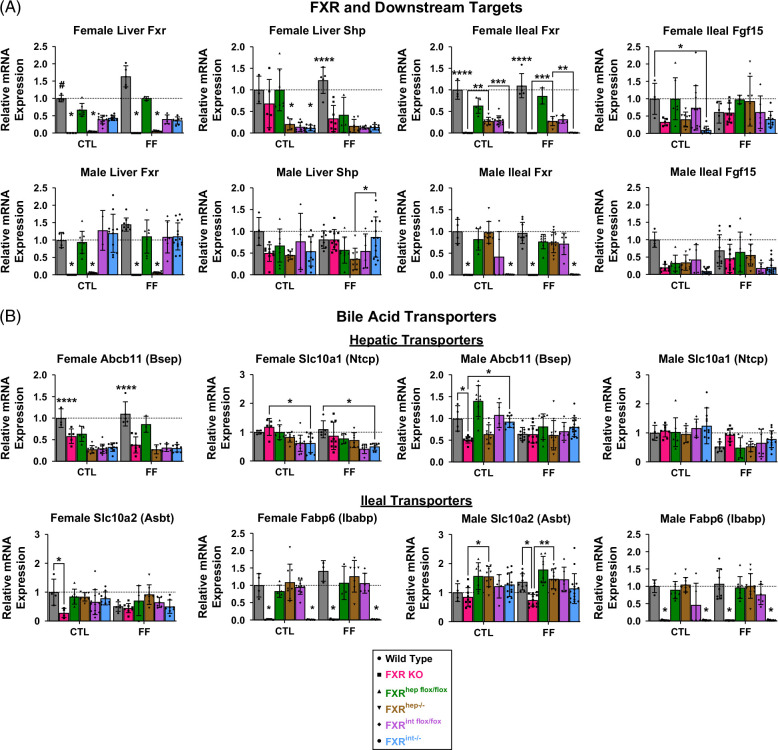

**Figure:**
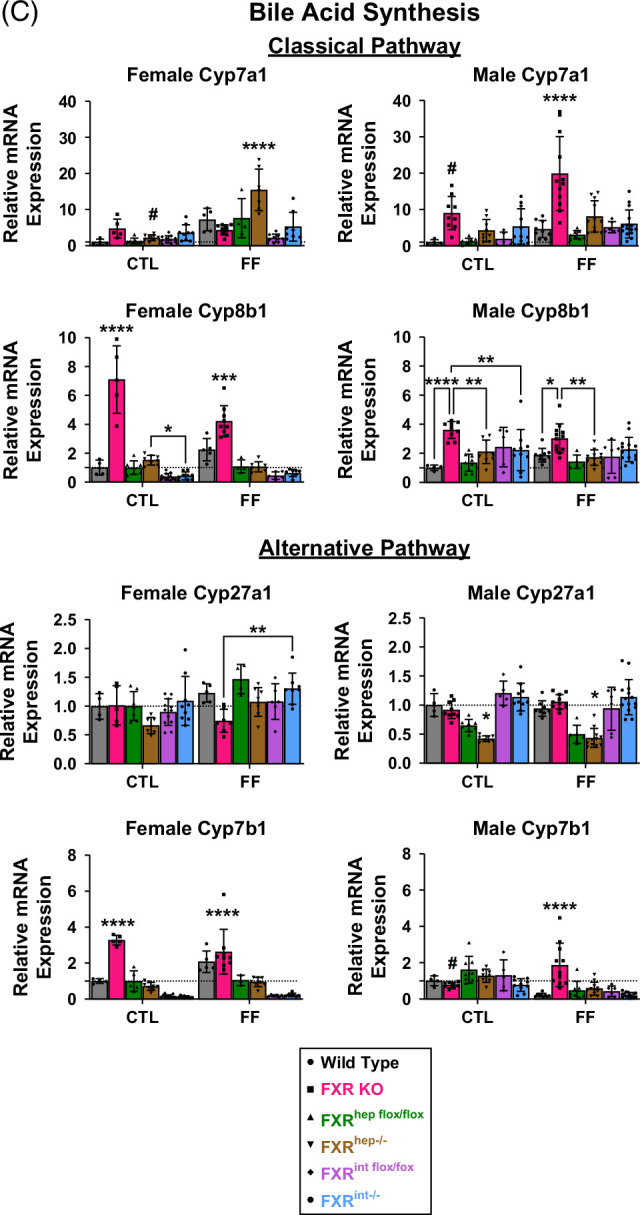

As previously mentioned, FXR inhibits BA synthesis by suppressing the mRNA expression of genes encoding key rate-limiting synthetic enzymes.^13^ To further examine FXR activity, relative mRNA expression was measured via qPCR for genes encoding for BA synthesis enzymes of both the classical and alternative pathways. Cyp7a1 mRNA levels were significantly increased in female FXR^hep−/−^ mice on the FF diet compared to all other groups (Figure 2C). In addition, female FXR^hep−/−^ mice on CTL diet displayed significantly increased expression of Cyp8b1 mRNA compared to the FXR^int−/−^ mice, demonstrating an upregulation of the classical BA pathway in these FXR^hep−/−^ mice. FXR KO female mice had increased expression of both Cyp8b1 and 25-hydroxycholesterol 7-alpha-hydroxylase (Cyp7b1) mRNA levels regardless of diet. No significant changes were seen for sterol-27-hydroxylase (Cyp27a1). These same trends were not seen in the male mice. Whole-body FXR inactivation led to an upregulation of the classical pathway in males, but there was no significance seen in tissue-specific KO groups.

In addition to BA synthesis enzymes, BA transporters are also regulated by FXR to maintain BA homeostasis.^33^ We evaluated the change in mRNA expression of BA influx and efflux transporters in the liver, such as sodium taurocholate co-transporting peptide (Slc10a1/Ntcp) and bile salt export pump (Abcb11/Bsep) (Figure 2B). Apical sodium-dependent bile acid transporter (Slc10a2/Asbt), ileal bile acid-binding protein (Fabp6/Ibabp), and organic solute transporters alpha (Ostα) and beta (Ostβ) were assessed in the ileum. No significant changes were seen in Bsep, Ntcp, or Asbt mRNA expression. Ibabp mRNA was depleted in the FXR KO and FXR^int−/−^ groups for both males and females. Ostα and Ostβ mRNAs were also significantly reduced in the FXR KO and FXR^int−/−^ groups for both sexes (Supplemental Figure S3, http://links.lww.com/HC9/B971).

### Deficiency of hepatic FXR leads to increased BA levels regardless of sex

To further assess the implications of BAs in the phenotypes expressed for each group, BA profiling was performed for the serum, liver (Tables 1 and 2; Supplemental Figures S4 and S5, http://links.lww.com/HC9/B971), and SI (Supplemental Table S2, http://links.lww.com/HC9/B971 and Supplemental Figure S6, http://links.lww.com/HC9/B971). The female FXR^hep−/−^ on the FF diet manifested the most total BAs overall among all groups, including the males, for each tissue. These mice also contained the highest percentage of tri-OH BAs [cholic acid (CA), and muricholic acids (MCAs)] and the lowest percentage of di-OH BAs [ursodeoxycholic acid (UDCA), (MDCA), chenodeoxycholic acid (CDCA), (HDCA), and deoxycholic acid (DCA)] in serum and liver. This group displayed the second highest levels for these same parameters in the intestine following the female FXR^hep−/−^ mice on CTL diet. In addition, these mice displayed the highest 1°/2° BA ratio. To assess Cyp8b1 activity, a proportion of CA to CDCA was calculated. The female FXR^hep−/−^ mice on CTL diet manifested the highest CA/CDCA ratio out of all groups, followed by the female FXR KO group on CTL diet for serum, liver, and SI. C4, a BA intermediate and indirect measure of Cyp7a1 activity in serum, was highest in the WT mice on FF diet regardless of sex (Supplemental Figure S7 http://links.lww.com/HC9/B971). For the male mice, the highest total BAs were measured in the FXR^hep−/−^ groups on both diets. CA/CDCA ratios were also highest in these mice. Overall, the female groups generally have more BAs compared to the male groups. In addition, the ratio of amidated/unamidated BAs is higher in the female mice compared to the males, including BAs like taurocholate.

**TABLE 1:** Serum BA indices

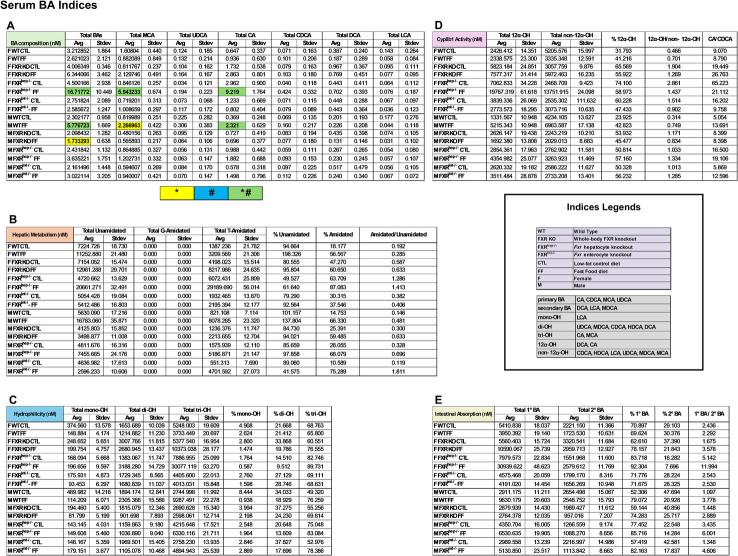

*Note*: Serum BA indices were calculated to address the following criteria: (A) BA composition, (B) hepatic metabolism, (C) hydrophilicity, (D) Cyp8b1 activity, and (E) intestinal absorption. (n=3–4).

*Represents significant difference between genotypes and within diet; ^#^Represents significant difference within genotype and across diet.

Abbreviation: BA, bile acid.

**TABLE 2:** Liver BA indices

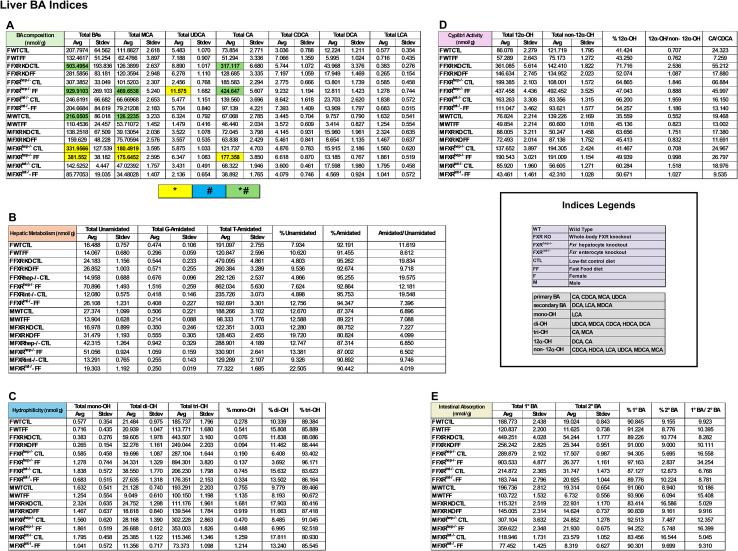

*Note*: Liver BA indices were calculated to address the following criteria: (A) BA composition, (B) hepatic metabolism, (C) hydrophilicity, (D) Cyp8b1 activity, and (E) intestinal absorption. (n=3–4).

*Represents significant difference between genotypes and within diet; ^#^Represents significant difference within genotype and across diet.

Abbreviation: BA, bile acid.

### Deletion of hepatic FXR increased inflammation and fibrosis in female mice

**FIGURE 3:**
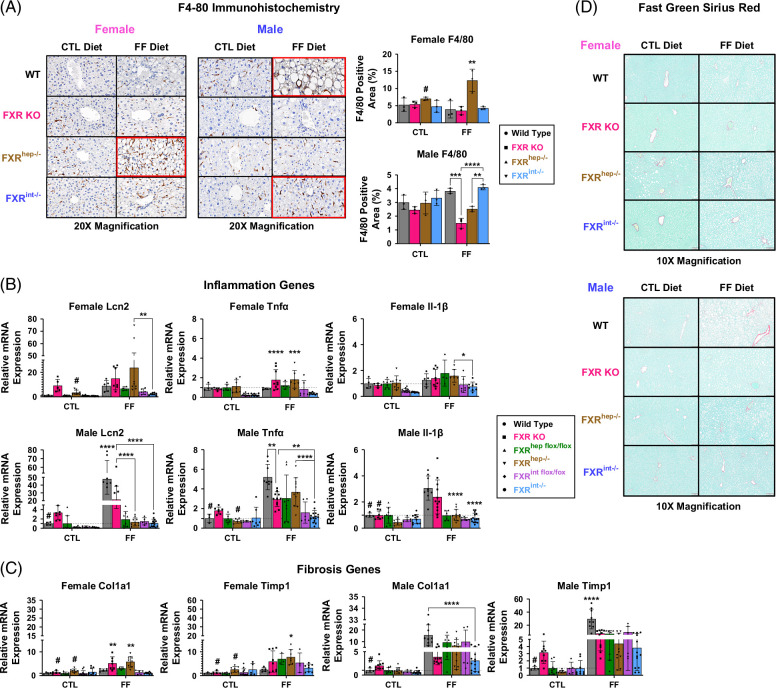
Inflammation and fibrosis. (A) Immunohistochemistry for F4/80 protein in female (left) and male (right) with corresponding semiquantitative analysis of % positive staining area for WT, FXR KO, FXR^hep−/−^, and FXR^int−/−^. (B) Inflammation, mRNA expression in females (top) and males (bottom) for genes: Lcn2, Tnfα, and Il-1β. (C) Fibrosis, mRNA expression in females (left) and males (right) for genes Col1a1 and Timp1. (D) Fast green Sirius red staining for females (top) and males (bottom). Data represented as mean±SD (n=4–20). Two-way ANOVA; *p<0.05, **p<0.01, ***p<0.001, and ****p<0.0001. *Represents significant difference between genotypes and within diet; ^#^Represents significant difference within genotype and across diet. Abbreviations: CTL, control; FF, fast food; WT, wild type.

We next assessed the impact of tissue-restricted FXR deletion on inflammation and fibrosis. In females, expression of genes involved in inflammation: lipocalin-2 (Lcn2), Tnfα, Il-6, and Il-1β, was significantly increased in FXR^hep−/−^ compared to FXR^int−/−^ mice, fed FF diet (Figure 3B). Even though significant differences were not found between the female FXR^hep−/−^ and FXR^int−/−^ mice on CTL diet, there was a trending decrease in expression of these inflammatory genes in the FXR^int−/−^ mice compared to the FXR^hep−/−^ mice, demonstrating higher basal inflammation in the FXR^hep−/−^ mice independent of the diet. Immunohistochemistry staining of F4/80, a general macrophage marker and indirect measure of inflammation, also showed increased macrophage presence in the FXR^hep−/−^ mice on FF Diet, suggesting increased inflammation (Figure 3A). In addition, collagen, type 1, alpha 1 (Col1α1) and tissue inhibitor of metalloproteinase 1 (Timp1), markers of liver fibrosis, were also significantly upregulated in the FXR^hep−/−^ mice, suggesting increased fibrosis (Figure 3C). Fast Green Sirius Red staining did not show changes in fibrosis across treatment groups (Figure 3D).

In contrast, for the male mice, the FF diet induced inflammation and fibrosis in the WT mice over the conditional FXR KO groups. This interesting finding was seen in both the IHC F4/80 staining and the relative mRNA expression data. Only on the FF diet were there significant increases seen in Col1α1 and Timp1 in male FXR^hep−/−^ mice compared to FXR^int−/−^.

### Intestinal FXR critical in suppressing lipid accumulation in the liver

**FIGURE 4:**
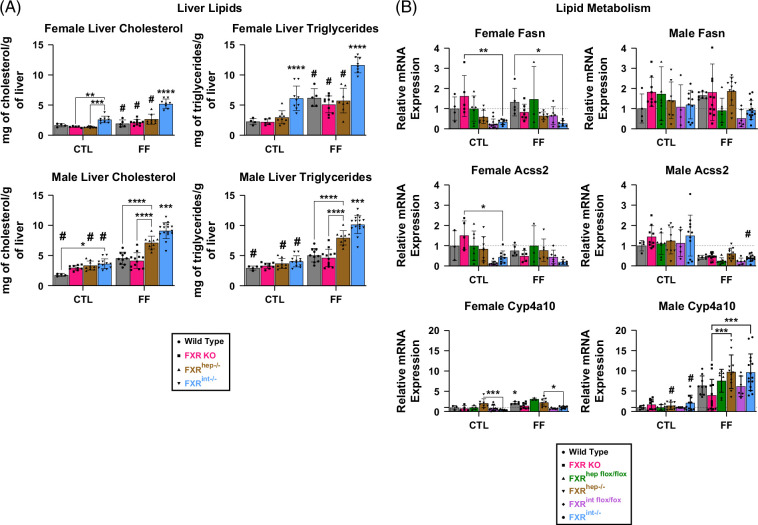
Lipid metabolism. (A) Liver triglycerides and cholesterol levels in female (top) and male (bottom) groups: WT, FXR KO, FXR^hep−/−^, and FXR^int−/−^. (B) Female (left) and male (right) lipid metabolism gene mRNA expression: Fasn, Acss2, and Cyp4a10. Data represented as mean±SD (n=4–20). Two-way ANOVA; *p<0.05, **p<0.01, ***p<0.001, and ****p<0.0001. *Represents significant difference between genotypes and within diet; ^#^Represents significant difference within genotype and across diet. Abbreviations: CTL, control; FF, fast food.

The expression of acyl-coA synthetase short chain family member 2 (Acss2), a gene that encodes the enzyme responsible for catalyzing the activation of acetate for use in lipid synthesis, was significantly increased in female FXR^hep−/−^ compared to FXR^int−/−^ mice, regardless of diet (Figure 4B). Fatty acid synthase (Fasn) was also examined, yet no significance was found, only a minor trending increase in FXR^hep−/−^ compared to FXR^int−/−^. Hepatic deletion of FXR resulted in a trending increase in the expression of Cyp4a10, a marker for β-oxidation, compared to FXR^int−/−^ mice. In contrast, unconcreted trends were seen in the male conditional FXR KO groups. Fasn was significantly increased in the male FXR^hep−/−^ FF mice compared to the FXR^int−/−^ FF group, while a trending increase was observed in the same groups for Acss2. No changes were seen in Cyp4a10.

To further assess lipid content, a liver lipid extraction assay was conducted (Figure 4A). We discovered that the FXR^int−/−^ mice on FF diet displayed significant amounts of cholesterol and triglycerides, independent of sex. In females, this significance was also independent of the diet. To further explore the connection between intestinal FXR deletion and lipid accumulation, ceramide metabolism was examined via qPCR (Figure 4C). Ceramides are intracellular signals for apoptosis, but also increase sterol regulatory element-binding protein 1c (Srebp-1c) in the liver, thereby promoting lipogenesis.^34^ Intestinal FXR has been shown to increase the expression of genes involved in ceramide synthesis.^35^ No changes were seen in the expression of ceramide synthase 2 (Cer2), serine palmitoyltransferase 2 (Sptlc), or sphingomyelin phosphodiesterase (Smpd3), ceramide synthesis genes (Supplemental Figure S8, http://links.lww.com/HC9/B971).

### IPA analysis suggests activation of novel neuro-related pathways

To explore potential underlying mechanisms contributing to the FXR tissue-specific differences seen, we submitted WT, FXR KO, FXR^hep−/−^, and FXR^int−/−^ liver and ileum RNA for RNA-seq analysis (Supplemental Figures S9 and S10, http://links.lww.com/HC9/B971). Due to our interest in FXR tissue-specificity, the presented results focus on the comparison of FXR^hep−/−^ and FXR^int−/−^ groups.

**FIGURE 5:**
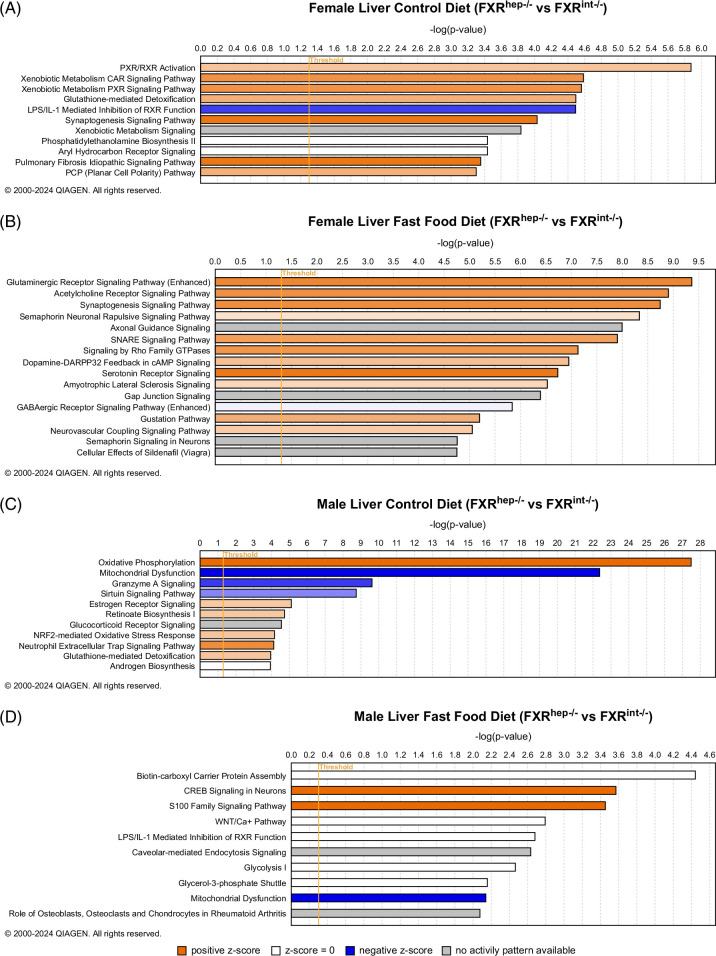
Ingenuity pathway analysis (IPA) in the liver (FXR^hep−/−^ vs. FXR^int−/−^). (A) Canonical pathways of liver samples from female FXR^hep−/−^ versus FXR^int−/−^ mice fed a control diet. (B) Canonical pathways of liver samples from female FXR^hep−/−^ versus FXR^int−/−^ mice fed fast food (FF) diet. (C) Canonical pathways of liver samples from male FXR^hep−/−^ versus FXR^int−/−^ mice fed a control diet. (D) Canonical pathways of liver samples from female FXR^hep−/−^ versus FXR^int−/−^ mice fed FF diet (n=3 per group).

In the liver, ingenuity pathway analysis (IPA) showed various activated canonical pathways (positive *z*-score) that are neuro-related signaling in the female FXR^hep−/−^ versus FXR^int−/−^ mice fed FF diet, for example, glutaminergic receptor signaling, acetylcholine receptor signaling, and serotonin receptor signaling, etc. This was further confirmed in the summary graph (Figures 5A, B). For the female FXR^hep−/−^ versus FXR^int−/−^ comparisons under the CTL diet, CAR and PXR signaling pathways were activated along with glutathione-mediated detoxification, while the LPS/IL-1–mediated inhibition of RXR function was the only negatively enriched pathway (negative *z*-score). Not many activated or inhibited pathways were detected in the male FF FXR^hep−/−^ versus FXR^int−/−^ groups (Figures 5C, D). One interestingly inhibited pathway was in relation to mitochondrial dysfunction, suggesting that the male FXR^hep−/−^ mice on FF diet have reduced mitochondrial dysfunction than the FXR^int−/−^ mice. These same mice on CTL diet also showed inhibited mitochondrial dysfunction pathway along with granzyme A signaling, a cell death mechanism. The oxidative phosphorylation pathway was the most significantly activated pathway.

**FIGURE 6:**
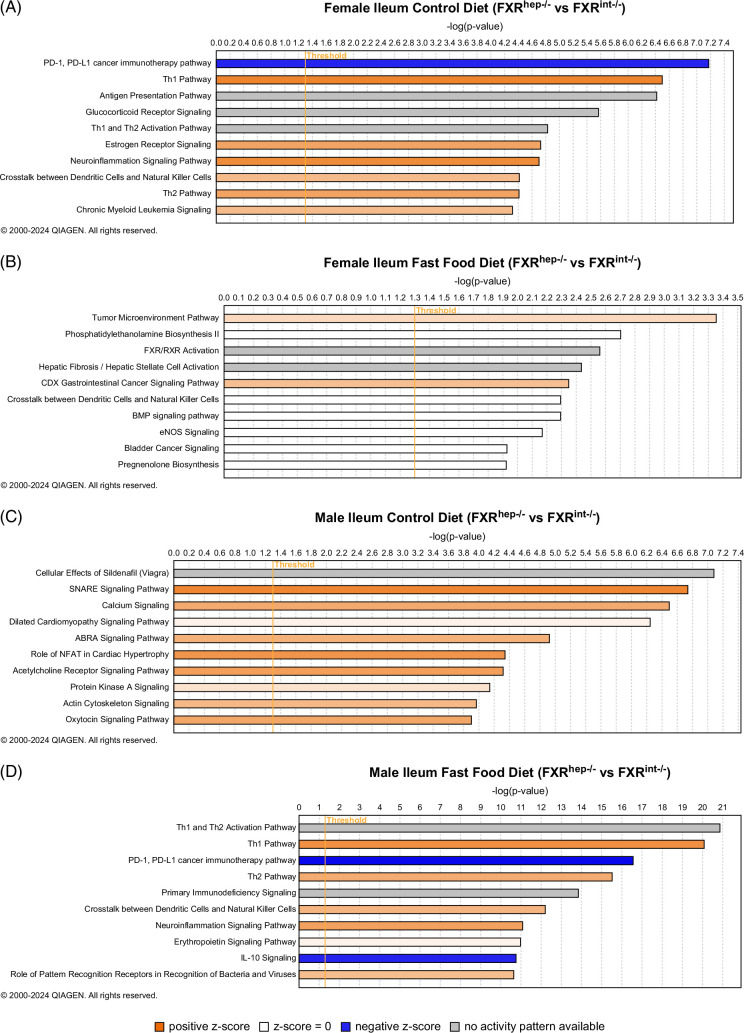
Ingenuity pathway analysis (IPA) in the ileum (FXR^hep−/−^ vs. FXR^int−/−^). (A) Canonical pathways of ileum samples from female FXR^hep−/−^ versus FXR^int−/−^ mice fed a control diet. (B) Canonical pathways of ileum samples from female FXR^hep−/−^ versus FXR^int−/−^ mice fed FF diet. (C) Canonical pathways of ileum samples from male FXR^hep−/−^ versus FXR^int−/−^ mice fed a control diet. (D) Canonical pathways of ileum samples from female FXR^hep−/−^ versus FXR^int−/−^ mice fed FF diet (n=3 per group).

In the ileum, IPA analysis showed that female FXR^hep−/−^ mice on FF diet enriched mostly the tumor/cancer pathways (Figure 6B). Specifically, the tumor microenvironment and gastrointestinal cancer pathways were activated compared to FXR^int−/−^ on FF diet. Various immune pathways were significantly activated in the CTL diet-fed female FXR^hep−/−^ mice compared to the FXR^int−/−^, including T-helper cells 1 and 2 (Figure 6A). As seen in the female liver IPA analysis, neuroinflammation was also significantly activated in the female FXR^hep−/−^ mice. PD-1/PD-L1 axis pathway, critical for immune response in the tumor microenvironment, is repressed in the female FXR^hep−/−^ mice on CTL diet. For males on this same diet, IPA displayed activation of various pathways, including calcium signaling, acetylcholine receptor signaling, and soluble N-ethylmaleimide-sensitive factor protein attachment protein receptor (SNARE) signaling pathways (Figure 6C). The FF diet, for the male ileum, shows similar significant canonical pathways as the females (Figure 6D). This included the activated pathways, such as neuroinflammation, T-helper cell 1 and 2, along with the repressed pathways such as the PD-1/PD-L1 axis and IL-10 signaling.

## DISCUSSION

For the past several years, various nuclear receptors and transcription factors have been studied as therapeutic targets for MASH, including FXR. FXR has proven to be an ideal therapeutic target for drug development for MASH, although off-target effects of nonspecific FXR agonists have halted hopeful therapeutics from being approved by the FDA. Selective modulation surrounding nuclear receptors, as a targeted approach, is not a new concept, particularly for type I receptors such as the estrogen receptor,^36^ yet, studies including selective modulation of FXR, especially liver-specific FXR modulation, are limited. Furthermore, studies that not only compare various FXR conditional KO models but also include female mice have not yet been reported. Filling this gap in knowledge is critical to answer important mechanistic questions for targeted drug development for MASLD/MASH.^37^

Although primarily expressed in the liver and ileum, FXR is ubiquitously expressed throughout the body, albeit at generally lower levels in other tissues. The question remains how to target FXR in certain cells or tissues. Previously, our group addressed the uncertainty of whether FXR even functions in a tissue-specific manner, where genome-wide ChIP-seq technologies were used to examine FXR-binding sites in mouse liver and intestines.^38^ There was only an 11% overlap between liver and intestinal FXR-binding sites in the mouse genome, suggesting underlying regulation of FXR tissue-specific functionality. Translating this work into an *in vivo* project using FXR KO mouse models, we further examined this concept in MASH.

Using various FXR KO mouse models fed a relevant MASH diet or control diet, we initially wanted to characterize the surface level phenotypes of each group to get a first glance at the FXR tissue-specific differences. The chosen MASH diet incorporates cholesterol to invoke lipotoxicity, inflammation,^39^ and reduce the time span of MASH development in mice from 6 to 4 months in lieu of a typical Western diet. With our initial work assessing parameters such as body weight change over time and serum biochemistry, the female FXR^hep−/−^ group displayed the most severe MASH phenotype. These mice were more resistant to weight gain, yet had the largest LW/BW ratios, along with a significant increase in circulating serum liver injury enzymes and serum lipids. Increases in ALT, cholesterol, and triglycerides were seen regardless of the diet, showing a remarkable phenotype of potential liver injury and fatty liver from hepatic FXR deletion alone. The female FXR^int−/−^ mice mimicked the control group and did not display increases in liver injury markers. In contrast, we found that male conditional FXR KO mice displayed altered phenotypes, with more severe potential liver injury observed in WT mice. This was the first sign of potential sex differences for FXR tissue-restricted modulation. A noteworthy finding from one of our previous studies,^15^ showed male FXR KO mice displaying higher injury following a pure high-fat diet without the addition of fructose, suggesting that FXR may be more critical in protecting the male livers from lipid-induced injury than fructose-induced injury.

To confirm our findings and further distinguish our models, we assessed each of the key characteristics for MASH: inflammation, fibrosis, and steatosis. In females, deletion of hepatic FXR led to increased expression of genes involved in inflammation (Lcn2, Tnfα, Il-1β) and fibrosis (Col1α1 and Timp1). Increased inflammation was further confirmed by F4/80 immunostaining. Fibrosis was not confirmed by Fast Green Sirius Red staining, as manifestation of fibrosis in C57BL/6J mice is difficult^40^ and 16 weeks is not enough time for the phenotype to develop, but is enough to see changes at the gene level. Again, we found the most severe injury in the female FXR^hep−/−^ mice and the least in the female FXR^int−/−^ mice. In fact, it seems as though intestinal deletion of FXR in female mice protects against liver injury. In addition, we found that the FXR^int−/−^ mice have lower baseline expression of these genes compared to their WT counterparts, further confirming this potential theory. Contrarily, increased expression of inflammation and fibrosis genes was detected in the male WT group on the MASH diet and not the conditional FXR KO models, suggesting that FXR deletion is perhaps more detrimental for inflammation and fibrosis in the female cohorts opposed to the males. Again, as above mentioned, this resistance to inflammation and fibrosis in FXR-deficient male mice may also reflect response differences to high-fat versus high fructose/sucrose challenges during MASH development, which may be sex dependent as well.

Regarding steatosis, liver lipid extraction results demonstrated the most abundant cholesterol and triglyceride levels found in the intestinal FXR KO groups, regardless of sex, and for the female mice, independent of diet, suggesting the critical nature of intestinal FXR in suppressing lipid accumulation in the liver. Studies have shown the distinct connection between intestinal FXR signaling and lipid accumulation in the liver.^34^^,^^35^ Ceramides not only act as intracellular signals for apoptosis, but also as inducers of *de novo* lipogenesis in the liver. Furthermore, genes involved in ceramide synthesis in the ileum, such as Smpd3 and Sptlc2, are direct downstream targets of intestinal FXR signaling^41^; therefore, when intestinal FXR signaling is depleted, these genes are downregulated, and hepatic steatosis is decreased. In our hands, when intestinal FXR signaling was depleted, Smpd3, Sptlc2, and Cers2 gene levels were comparable to normalized levels in the WT groups, and liver lipids were significantly increased. Although ceramide levels were not decreased with the lack of intestinal FXR signaling, they were not increased, suggesting the role of another potential mechanism independent of ceramide signaling that may be contributing to the increased lipid bioavailability in the livers of the FXR^int−/−^ groups, such as Fgf15/FGF19 signaling. In fact, mice treated with human FGF19 and fed a high-fat diet displayed increased liver fatty acid oxidation and depletion of local liver triglycerides. In addition, our group has previously shown that Fgf15 deficiency prevents a compensatory decrease of lipid synthesis genes, Fasn and Acss2, seen with high-fat feeding.^42^ These studies demonstrate the importance of Fgf15/FGF19 signaling in mitigating hepatic steatosis through the possible suppression of gut lipid absorption.

Overall, there have been clear sex differences observed in this study, with the female FXR^hep−/−^ mice having the more severe degrees of MASH and liver injury compared to the FXR^int−/−^ mice. The male mice showed mostly unconcreted trends when comparing FXR^hep−/−^ versus FXR^int−/−^. Sex differences in disease models for liver pathologies are not a new discovery. In fact, research has shown female mice are more susceptible to liver injury in cholestatic disease models compared to male mice.^43^^,^^44^ The obvious factor is hormonal differences, with the abundance of estrogen in female mice over male mice. Estrogen is known to induce cholestasis^45^^,^^46^ and the contributing mechanisms have been evaluated, including blockage of BSEP and increased production of BAs, especially during the late stage of gestation.^47^ In addition, sulfated progesterone metabolites are known to directly impair biliary transport of BAs.^48^ Thus, the influence of hormones is pivotal when considering sex differences pertaining to BA metabolism.

BAs also play a major role in sex differences in liver pathologies, including cholestasis and fibrosis, as the multidrug resistance protein 2 (Mdr2) KO female mice have a more hydrophobic BA pool and higher levels of taurocholate in the bile when compared to their male counterparts, which is associated with more severe liver injury.^43^ Generally, hydrophobic BAs are more toxic than hydrophilic.^49^ The increased ALP values in the female FXR^hep−/−^ indicate cholestasis in these mice, further contributing to their overall liver injury and susceptibility. Our BA profiling findings further confirmed cholestasis in the female FXR^hep−/−^, particularly the FF-fed mice, due to these mice manifesting the highest levels of total BAs compared to all groups, including the males. Furthermore, this group displayed the highest percentage of tri-OH BAs and the lowest percentage of di-OH BAs, indicating a relatively hydrophilic BA pool. As seen with the Mdr2 KO mice, taurocholate was highest in the female mice compared to the males, and overall amidated/unamidated ratios were higher. Although the male mice generally expressed lower levels of BAs overall compared to the female, the FXR^hep−/−^ groups, regardless of diet, had the highest total BAs among the other male genotypes, demonstrating the critical nature of hepatic FXR in regulating BA synthesis. Yet, with high levels of BAs, these mice were still more resistant to liver injury overall compared to the female mice.

RNA sequencing was performed in hopes of elucidating the underlying mechanisms contributing to the sex-specific and tissue-specific differences observed in FXR modulation in MASH. General bulk RNA sequencing is very exploratory in nature, and although our group would have benefited more from a single-cell analysis approach, these findings have laid a hopeful foundation for future studies to expand upon. In our findings, there seems to be a connection between hepatic FXR deletion and neuro-related pathways such as neuroinflammation, likely a result of altered FXR signaling and BA dysregulation, which have been implicated in various neurological disorders and diseases.^50^ Detection of these activated canonical pathways within the female FXR^hep−/−^ mice in comparison to the FXR^int−/−^ mice potentially highlights not only the critical nature of hepatic FXR in preserving liver health, but also neuronal health. These findings need to be followed up in the future with protein expression and functional analysis. The IPA findings also provide insight into the lack of injury seen in the male FXR^hep−/−^ versus FXR^int−/−^ mice with the inhibition of the mitochondrial dysfunction canonical pathway. Endoplasmic reticulum stress and mitochondrial dysfunction are recognized hallmarks in MASH etiology; thus, this further supports our other findings displaying the lack of injury for this model in the male cohort.

In conclusion, this work determined the tissue-specific functionality of FXR in mice for the development of MASH, with the female mice showing the most susceptibility to MASH development following liver-specific FXR deficiency. While the exact mechanisms contributing to these tissue-specific and sex-specific differences are still not well elucidated, further studies are needed to uncover mechanisms that are responsible for the selective modulation of FXR.

## Supplementary Material
