# Supplementary material for: Deletion of hepatic FXR leads to more severe MASH development in female mice
Source: Hepatol Commun. 2025 May 16;9(6):e0693. doi: 10.1097/HC9.0000000000000693 (PMC12088634; doi:10.1097/HC9.0000000000000693)
Supplement: Supplementary file 1 [file hc9-9-e0693-s001.pdf]

Supplementary Figures and Tables

Supplementary Table 1. List of Primers

	Forward Sequence	Reverse Sequence
**Acss2**	AAACACGCTCAGGGAAAATCA	ACCGTAGATGTATCCCCCAGG
**Asbt**	TTGCACAGCACAAGCAGTGA	TGCATTGAAGTTGCTCTCAGGT
**Bsep**	GCAGAAGCAAAGGGTAGCCATC	GGTAGCCATGTCCAGAAGCAG
**Cers2**	AAGTGGGAAACGGAGTAGCG	ACAGGCAGCCATAGTCGTTC
**Col1a1**	GAGAGAGCATGACCGATGGATT	TGTAGGCTACGCTGTTCTTGCA
**Cyp27a1**	GCTTTCTCTTCCCCAAG	CAGCCTCTTTCTTCCTCA
**Cyp4a10**	TTCCCTGATGGACGCTCTTTA	GCAAACCTGGAAGGGTCAAAC
**Cyp7a1**	AGCAACTAAACAACCTGCCAGTACTA	GTCCGGATATTCAAGGATGCA
**Cyp7b1**	CAGCTATGTTCTGGGCAATG	TCGGATGATGCTGGAGTATG
**Cyp8b1**	AGTACACATGGACCCCGACATC	GGGTGCCATCCGGGTTGAG
**Fasn**	GCTGCGGAAACTTCAGGAAAT	AGAGACGTGTCACTCCTGGACTT
**Fgf15**	GAGGACCAAAACGAACGAAATT	ACGTCCTTGATGGCAATCG
**Fxrα**	TCCGGACATTCAACCATCAC	TCACTGCACATCCCAGATCTC
**Ibabp**	GGTCTTCCAGGAGACGTGAT	ACATTCTTTGCCAATGGTGA
**IL-1β**	AAGGGCTGCTTCCAAACCTTTGAC	ATACTGCCTGCCTGAAGCTCTTGT
**Lcn2**	AATGTCACCTCCATCCTGGTCA	CCACTTGCACATTGTAGCTCT
**Ntcp**	GGCCACAGACACTGCGCT	AGTGAGCCTTGATCTTGCTGAACT
**Ostα**	GTCTCAAGTGATGAACTGCCA	TTGAGTGCTGAGTCCAGGTC
**Ostβ**	GTATTTTCGTGCAGAAGATGCG	TTTCTGTTTGCCAGGATGCTC
**Rps29**	GGTCACCAGCAG	GTCCAACTTAAT
**Shp**	CGATCCTCTTCAACCCAGATG	AGGGCTCCAAGACTTCACACA
**Smpd3**	CCTGACCAGTGCCATTCTTT	AGAAACCCGGTCCTCGTACT
**Srebp1c**	GGAGCCATGGATTGCACATT	GCTTCCAGAGAGGAGGCCAG
**Sptlc2**	TCACCTCCATGAAGTGCATC	CAGGCGTCTCCTGAAATACC
**Timp1**	GAGACCACCTTATACCAGCGT	CTGGGACTTGTGGGCATATC
**Tnfα**	ATGGCCTCCCTCTCATCAGT	GCTCCTCCACTTGGTGGTTT

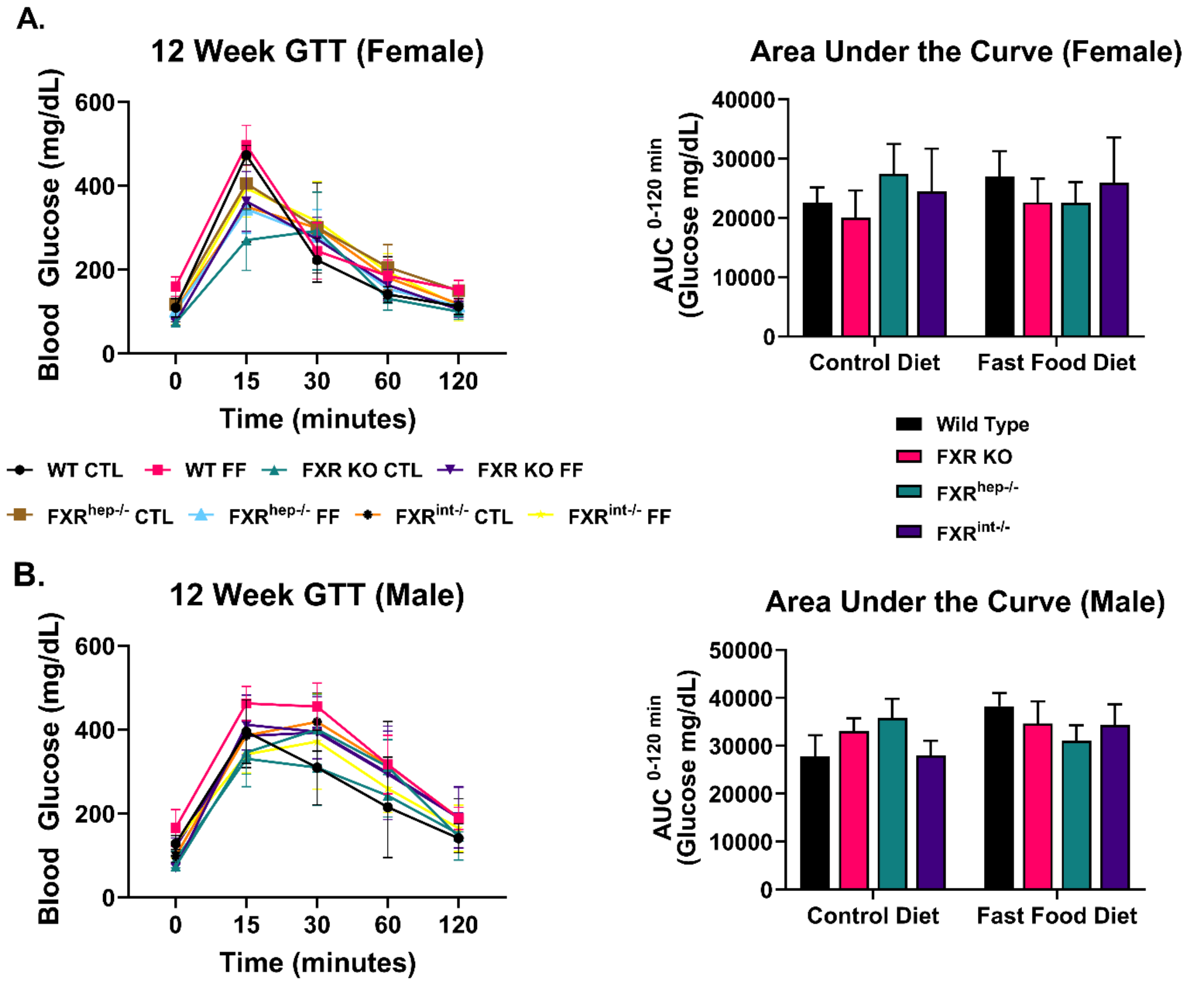

Supplementary Figure 1. Glucose Tolerance Test. GTT was conducted at week 12. A.) Female glucose tolerance curves and area under the curve B.) Male glucose tolerance curves and area under the curve. Data represented as the mean ± SD (n = 4-16). Two-way ANOVA. *p < 0.05, **p<0.01, ***p<0.001, ****p<0.0001. * Represents significant difference between genotypes and within diet; # Represents significant difference within genotype and across diet.

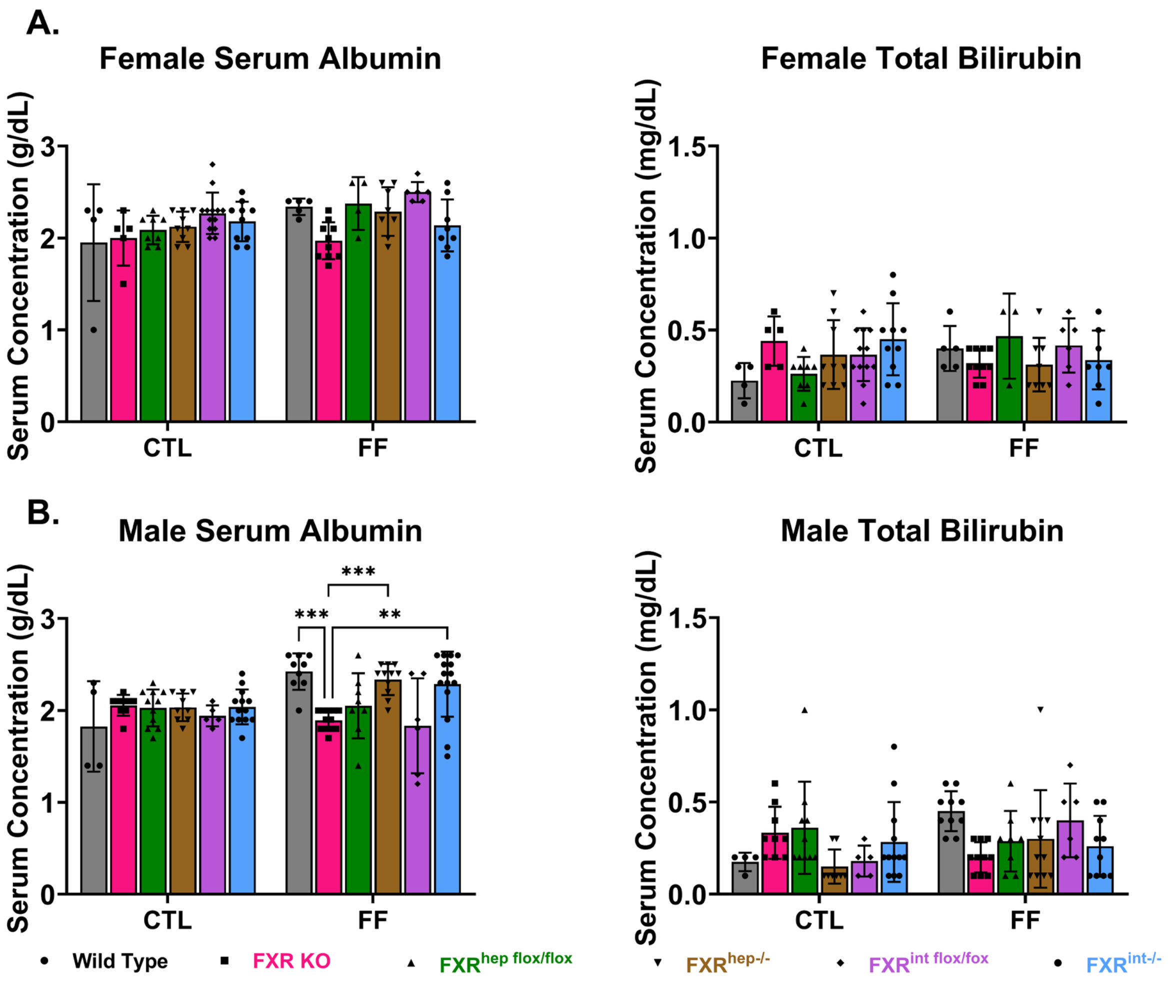

Supplementary Figure 2. Serum Albumin and Total Bilirubin. A.) Female serum albumin (left) and total bilirubin (right) and B.) Male serum albumin (left) and total bilirubin (right). Data represented as mean ± SD (n = 4-20). Two-way ANOVA. *p < 0.05, **p<0.01, ***p<0.001, ****p<0.0001. * Represents significant difference between genotypes and within diet; # Represents significant difference within genotype and across diet.

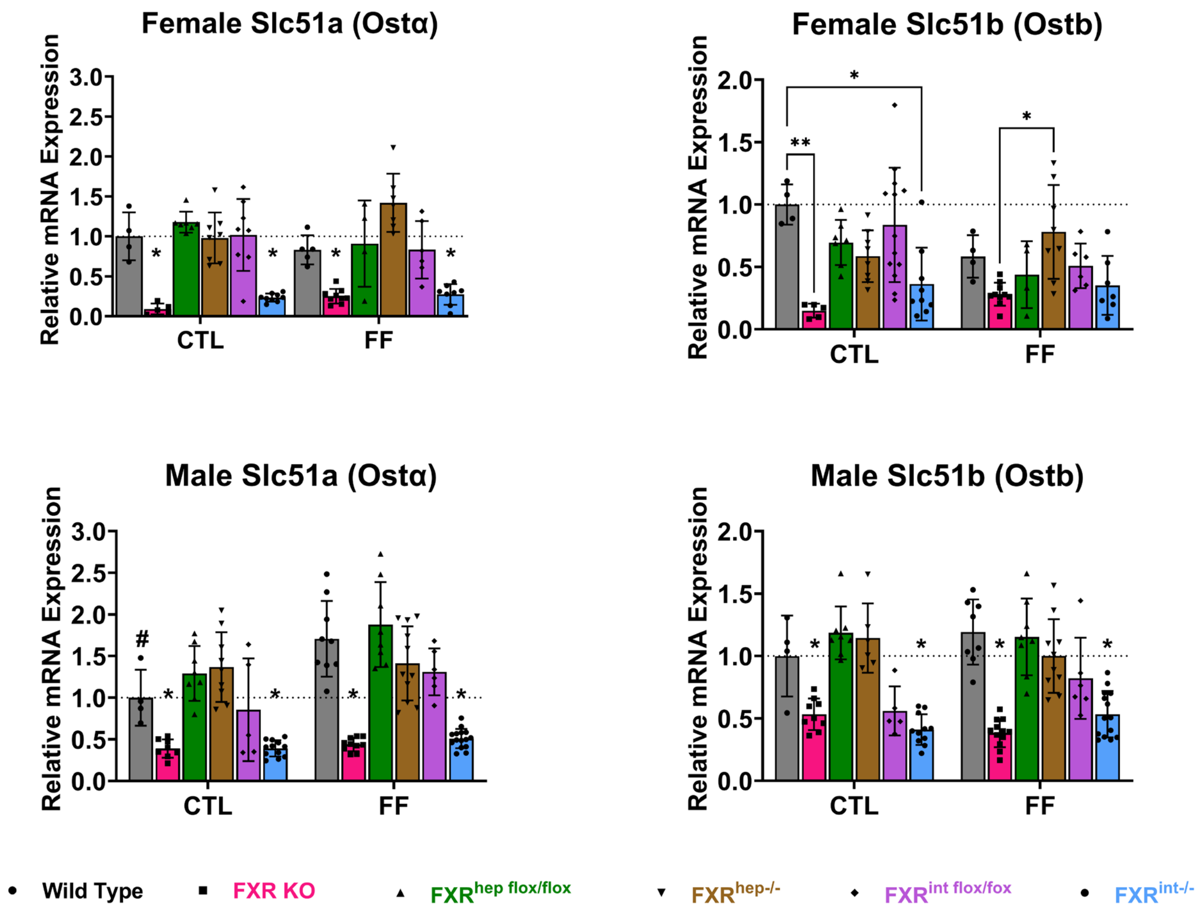

Supplementary Figure 3. Ostα/β mRNA Expression in the Ileum. Female Ostα (Slc51a) (top left) and Ostβ (Slc51b) (top right) and Male Ostα (Slc51a) (bottom left) and Ostβ (Slc51b) (bottom right). Data represented as mean ± SD (n = 4-20). Two-way ANOVA. *p < 0.05, **p<0.01, ***p<0.001, ****p<0.0001. * Represents significant difference between genotypes and within diet; # Represents significant difference within genotype and across diet.

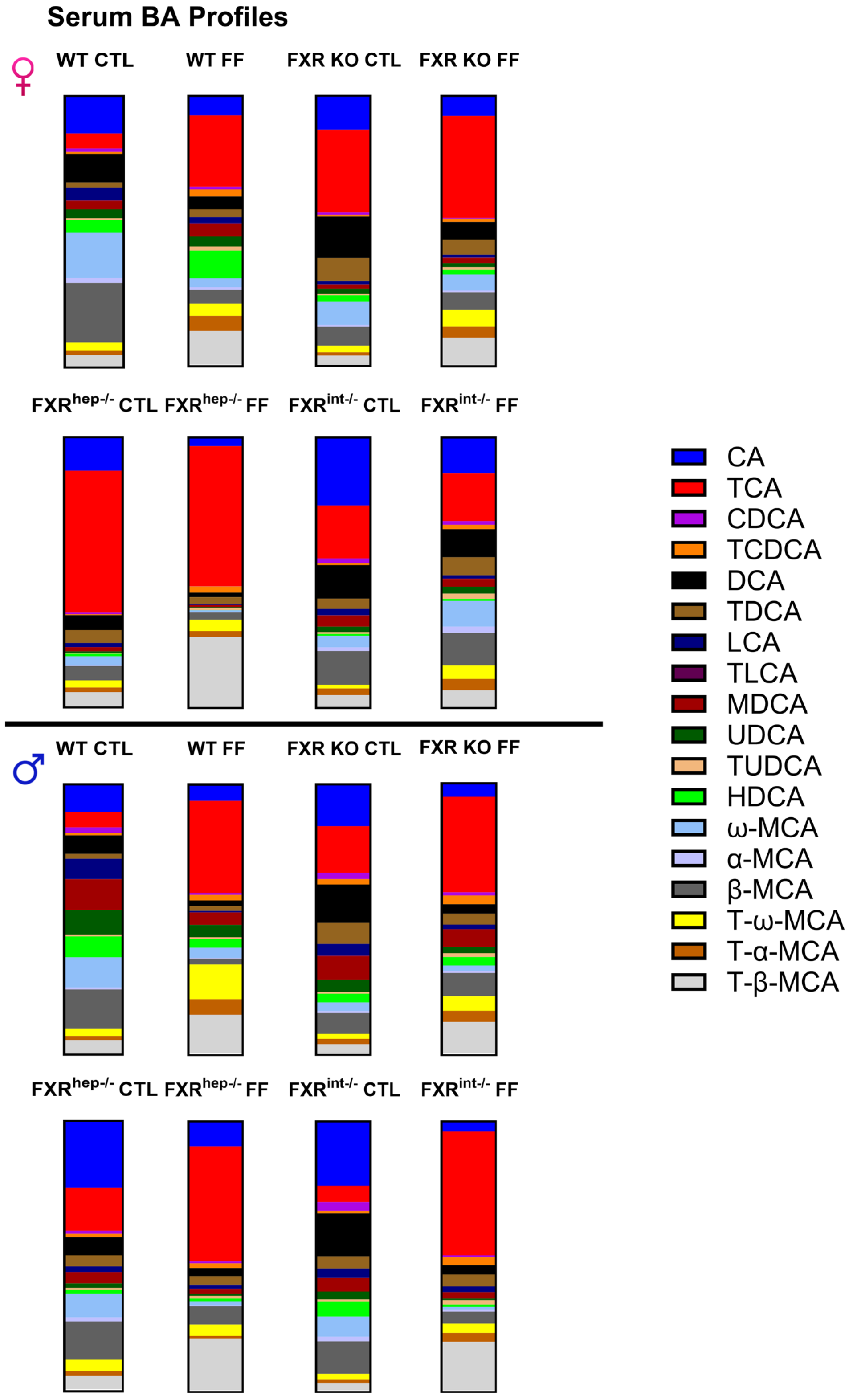

Supplementary Figure 4. Serum BA Profiles. Female (top) and male (bottom) serum individual BA species relative abundance. (n = 3-4)

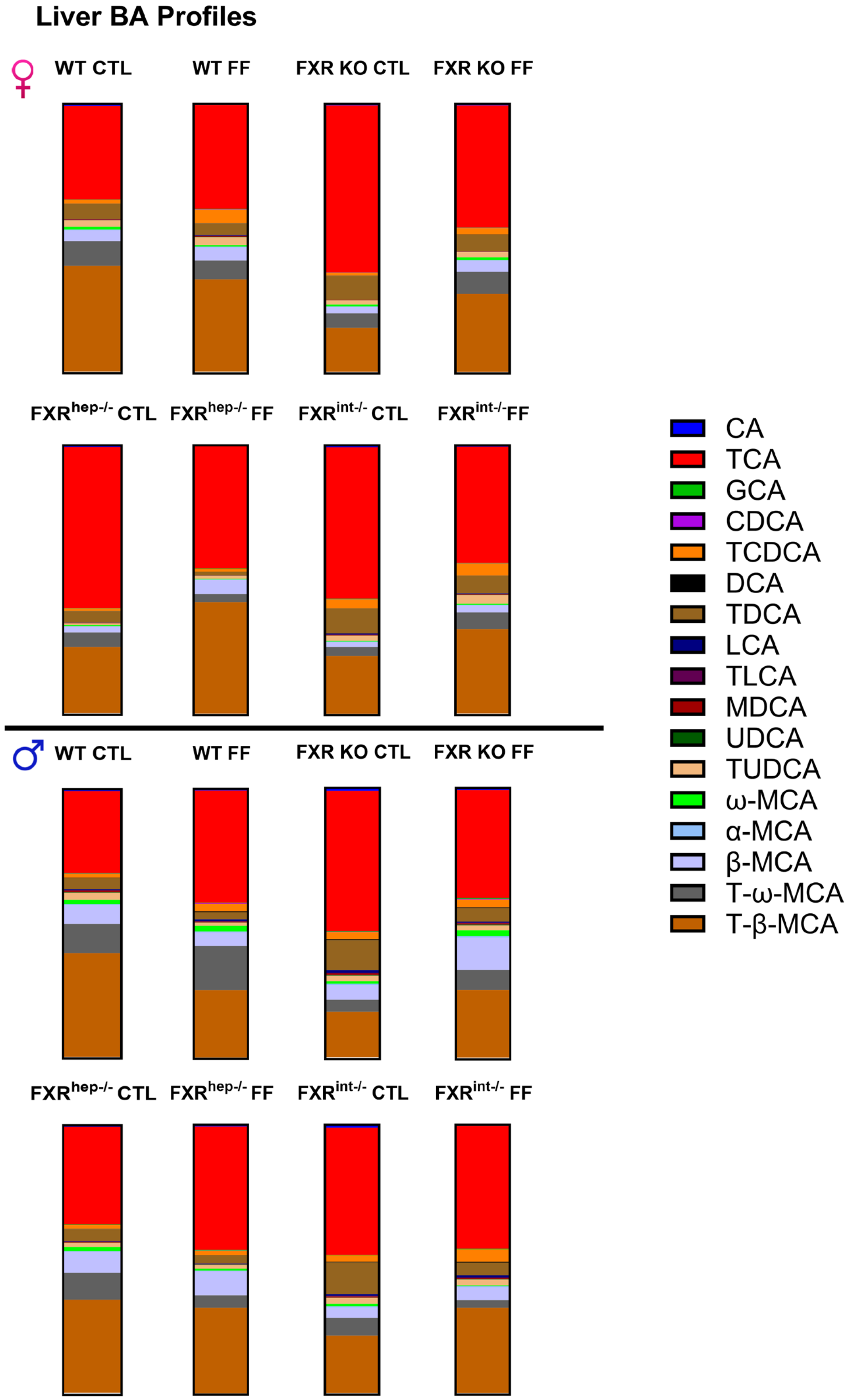

Supplementary Figure 5. Liver BA Profiles. Female (top) and male (bottom) liver individual BA species relative abundance. (n = 3-4)

Small Intestine BA Indices

A

BA composition (nmol/g)	Total BAs		Total MCA		Total UDCA		Total CA		Total CDCA		Total DCA		Total LCA	
	Avg	Stdev	Avg	Stdev	Avg	Stdev	Avg	Stdev	Avg	Stdev	Avg	Stdev	Avg	Stdev
FWTCTL	8565.834	1360.142	4311.675 (*)	11.202	190.782	4.439	3552.152 (*)	16.357	152.025	3.787	237.656	6.477	29.553	3.639
FWTFF	11992.94 (*#)	2394.632	6260.666 (*#)	43.837	414.369	9.990	4625.914	28.545	429.649	9.308	186.097	6.000	44.567	1.784
FFXRKOCTL	8891.414	1104.560	1802.24	11.330	107.145	5.063	6335.745	26.905	117.670	5.877	447.622	10.277	41.255	1.290
FFXRKOFF	10280.34 (#)	1005.546	3597.143 (#)	12.415	201.539	5.987	5907.622	29.454	180.535	6.857	323.997	7.849	40.832	1.213
FFXR$^{hep-/-}$ CTL	10113.81 (*)	2644.814	2901.282	15.154	56.352	3.424	6813.340	26.598	66.824	2.812	216.692	6.996	26.018	2.896
FFXR$^{hep-/-}$ FF	12998.79 (*#)	1381.543	5761.848 (#)	12.026	152.564	5.656	6665.333	24.731	225.173	6.700	136.193	4.601	38.349	4.306
FFXR$^{int-/-}$ CTL	7923.35	244.932	2653.384	16.811	134.163	6.344	4570.619	24.219	191.016	8.300	308.859	12.355	33.213	1.889
FFXR$^{int-/-}$ FF	10476.74 (#)	1086.840	4117.82 (#)	14.932	304.558	8.522	5304.452	25.830	326.338	9.807	365.606	8.546	27.149	2.931
MWTCTL	6703.833 (#)	789.398	3626.487 (*)	11.216	163.673	4.492	2417.817	12.535	184.372	5.404	140.901	4.345	28.195	3.021
MWTFF	8672.965	789.670	4734.973	10.067	118.812	3.647	3504.008	13.403	177.795	4.802	65.746	2.682	28.441	3.132
MFXRKOCTL	7580.364	1504.346	2086.687	13.351	115.221	5.571	4628.506	28.109	138.263	6.518	524.097	17.205	31.381	3.220
MFXRKOFF	13010.89 (*#)	2579.177	5532.257 (*#)	16.347	231.619	5.811	6539.943 (*#)	26.772	348.538	8.222	292.278	7.396	32.319	1.465
MFXR$^{hep-/-}$ CTL	8564.847 (*)	1137.966	3319.414	11.764	121.568	4.230	4570.770	22.918	121.773	4.261	324.388	8.602	24.227	2.784
MFXR$^{hep-/-}$ FF	9058.768	1127.562	4430.865	13.938	120.824	3.190	4150.522	29.595	148.967	6.587	151.481	8.028	29.104	1.259
MFXR$^{int-/-}$ CTL	6174.947	1196.509	1840.526	13.386	131.345	5.453	3469.356	21.966	160.756	6.990	405.493	8.566	29.701	2.021
MFXR$^{int-/-}$ FF	8135.804 (#)	1174.237	3344.85 (#)	10.945	194.816	5.867	4130.382	17.190	320.085	5.845	189.394	3.933	25.872	2.829

*	#	*#

D

Cyp8b1 Activity (nmol/g)	Total 12α-OH		Total non-12α-OH		% 12α-OH	12α-OH/non- 12α-OH	CA/CDCA
	Avg	Stdev	Avg	Stdev			
FWTCTL	3789.809	13.316	4776.025	7.936	44.243	0.794	23.366
FWTFF	4812.011	22.553	7180.925	13.012	40.124	0.670	10.767
FFXRKOCTL	6783.366	22.042	2108.047	8.125	76.291	3.218	53.843
FFXRKOFF	6231.619	23.349	4048.724	8.942	60.617	1.539	32.723
FFXR$^{hep-/-}$ CTL	7030.032	21.048	3083.782	10.170	69.509	2.280	101.960
FFXR$^{hep-/-}$ FF	6801.526	19.376	6197.262	9.864	52.324	1.098	29.601
FFXR$^{int-/-}$ CTL	4879.478	20.323	3043.871	11.759	61.584	1.603	23.928
FFXR$^{int-/-}$ FF	5670.058	20.725	4806.685	11.030	54.120	1.180	16.254
MWTCTL	2558.718	10.091	4145.115	8.116	38.168	0.617	13.114
MWTFF	3569.753	10.091	5103.212	7.161	41.160	0.700	19.708
MFXRKOCTL	5152.603	24.124	2427.762	9.518	67.973	2.122	33.476
MFXRKOFF	6832.220	21.258	6178.674	11.444	52.512	1.106	18.764
MFXR$^{hep-/-}$ CTL	4895.158	18.567	3669.689	8.273	57.154	1.334	37.535
MFXR$^{hep-/-}$ FF	4302.003	23.480	4756.765	9.564	47.490	0.904	27.862
MFXR$^{int-/-}$ CTL	3874.849	19.202	2300.098	10.042	62.751	1.685	21.582
MFXR$^{int-/-}$ FF	4319.775	13.532	3816.029	8.526	53.096	1.132	12.904

B

Hepatic Metabolism (nmol/g)	Total Unamidated		Total G-Amidated		Total T-Amidated		% Unamidated	% Amidated	Amidated/Unamidated
	Avg	Stdev	Avg	Stdev	Avg	Stdev			
FWTCTL	2668.804	8.989	16.586	5.291	5883.874	11.881	31.156	68.884	2.211
FWTFF	1358.837	10.877	11.175	3.960	10630.380	21.253	11.330	88.732	7.831
FFXRKOCTL	1684.348	10.570	1.817	2.361	7209.230	16.127	18.944	81.101	4.281
FFXRKOFF	1410.504	11.078	8.070	4.593	8864.248	17.655	13.720	86.304	6.290
FFXR$^{hep-/-}$ CTL	1421.147	6.332	19.280	6.757	8680.902	20.077	14.052	86.023	6.122
FFXR$^{hep-/-}$ FF	1808.508	9.819	9.608	1.175	11184.749	16.509	13.913	86.118	6.190
FFXR$^{int-/-}$ CTL	1924.372	11.355	15.768	2.811	6010.307	18.805	24.287	76.055	3.131
FFXR$^{int-/-}$ FF	1708.579	10.954	11.815	3.356	8761.173	17.787	16.308	83.738	5.135
MWTCTL	2338.407	8.792	11.975	4.629	4360.489	8.523	34.882	65.223	1.870
MWTFF	2345.238	6.096	5.606	1.115	6328.354	10.381	27.041	73.031	2.701
MFXRKOCTL	1608.243	11.198	10.968	1.676	6188.705	20.180	21.216	81.786	3.855
MFXRKOFF	551.455	6.595	8.219	1.551	12456.900	21.266	4.238	95.805	22.604
MFXR$^{hep-/-}$ CTL	2607.319	9.787	15.803	3.198	5944.556	14.277	30.442	69.591	2.286
MFXR$^{hep-/-}$ FF	1582.293	11.696	6.380	1.351	7475.139	17.807	17.467	82.589	4.728
MFXR$^{int-/-}$ CTL	2391.088	9.270	11.220	2.608	3776.011	15.680	38.722	61.332	1.584
MFXR$^{int-/-}$ FF	541.878	6.461	7.836	4.099	7588.277	13.576	6.660	93.366	14.018

Indices Legends

WT	Wild Type
FXR KO	Whole-body FXR knockout
FXR$^{hep-/-}$	*Fxr* hepatocyte knockout
FXR$^{int-/-}$	*Fxr* enterocyte knockout
CTL	Low-fat control diet
FF	Fast Food diet
F	Female
M	Male

primary BA	CA, CDCA, MCA, UDCA
secondary BA	DCA, LCA, MDCA
mono-OH	LCA
di-OH	UDCA, MDCA, CDCA, HDCA, DCA
tri-OH	CA, MCA
12α-OH	DCA, CA
non- 12α-OH	CDCA, HDCA, LCA, UDCA, MDCA, MCA

C

Hydrophilicity (nmol/g)	Total mono-OH		Total di-OH		Total tri-OH		% mono-OH	% di-OH	% tri-OH
	Avg	Stdev	Avg	Stdev	Avg	Stdev			
FWTCTL	29.553	3.639	623.689	4.909	7863.827	13.147	0.345	7.281	91.805
FWTFF	44.567	1.784	1048.866	8.111	10886.579	22.204	0.372	8.746	90.775
FFXRKOCTL	41.255	1.290	693.904	7.087	8137.985	22.204	0.464	7.804	91.526
FFXRKOFF	40.832	1.213	718.881	6.593	9504.765	19.797	0.397	6.993	92.456
FFXR$^{hep-/-}$ CTL	26.018	2.896	358.773	4.546	9714.622	19.721	0.257	3.547	96.053
FFXR$^{hep-/-}$ FF	38.349	4.306	524.282	5.263	12427.181	18.370	0.295	4.033	95.603
FFXR$^{int-/-}$ CTL	33.213	1.889	652.177	8.757	7224.003	19.594	0.419	8.231	91.174
FFXR$^{int-/-}$ FF	27.149	2.931	1014.526	8.334	9422.272	19.262	0.259	9.684	89.935
MWTCTL	28.195	3.021	545.888	4.812	6044.304	11.672	0.421	8.143	90.162
MWTFF	28.441	3.132	386.702	3.799	8238.980	11.289	0.328	4.459	94.996
MFXRKOCTL	31.381	3.220	804.113	10.323	6715.193	19.550	0.414	10.608	88.587
MFXRKOFF	32.319	1.465	887.662	6.788	12072.199	20.422	0.248	6.822	92.785
MFXR$^{hep-/-}$ CTL	24.227	2.784	594.750	5.741	7890.184	16.350	0.283	6.944	92.123
MFXR$^{hep-/-}$ FF	29.104	1.259	437.220	5.749	8581.387	20.530	0.321	4.826	94.730
MFXR$^{int-/-}$ CTL	29.701	2.021	747.175	6.571	5309.882	16.742	0.481	12.100	85.991
MFXR$^{int-/-}$ FF	25.872	2.829	717.353	5.013	7475.232	13.902	0.318	8.817	91.881

E

Intestinal Absorption (nmol/g)	Total 1° BA		Total 2° BA		% 1° BA	% 2° BA	1° BA/ 2° BA
	Avg	Stdev	Avg	Stdev			
FWTCTL	8015.852	12.001	549.982	4.795	93.579	6.421	14.575
FWTFF	11316.228	20.472	676.708	6.156	94.357	5.643	16.722
FFXRKOCTL	8255.655	16.545	635.758	6.085	92.850	7.150	12.986
FFXRKOFF	9685.300	18.145	595.043	5.290	94.212	5.788	16.277
FFXR$^{hep-/-}$ CTL	9781.446	17.878	332.368	4.363	96.714	3.286	29.430
FFXR$^{hep-/-}$ FF	12652.354	16.833	346.433	3.913	97.335	2.665	36.522
FFXR$^{int-/-}$ CTL	7415.019	18.073	508.331	7.138	93.584	6.416	14.587
FFXR$^{int-/-}$ FF	9748.610	17.918	728.133	6.318	93.050	6.950	13.388
MWTCTL	6228.676	10.806	475.156	4.282	92.912	7.088	13.109
MWTFF	8416.775	10.415	256.190	3.127	97.046	2.954	32.854
MFXRKOCTL	6853.456	17.861	726.909	9.495	90.411	9.589	9.428
MFXRKOFF	12420.737	18.802	590.158	5.052	95.464	4.536	21.046
MFXR$^{hep-/-}$ CTL	8011.957	14.901	552.890	5.472	93.545	6.455	14.491
MFXR$^{hep-/-}$ FF	8730.353	18.759	328.414	4.555	96.375	3.625	26.583
MFXR$^{int-/-}$ CTL	5470.638	15.340	704.309	6.362	88.594	11.406	7.767
MFXR$^{int-/-}$ FF	7795.317	12.819	340.488	3.964	95.815	4.185	22.895

Supplementary Table 2. Small Intestine BA Indices. SI BA indices were calculated to address the following criteria: A.) BA composition, B.) hepatic metabolism, C.) hydrophilicity, D.) Cyp8b1 activity, and E.) intestinal absorption. Data in table are presented as mean ± SD (n=3-4). * Represents significant difference between genotypes and within diet; # Represents significant difference within genotype and across diet.

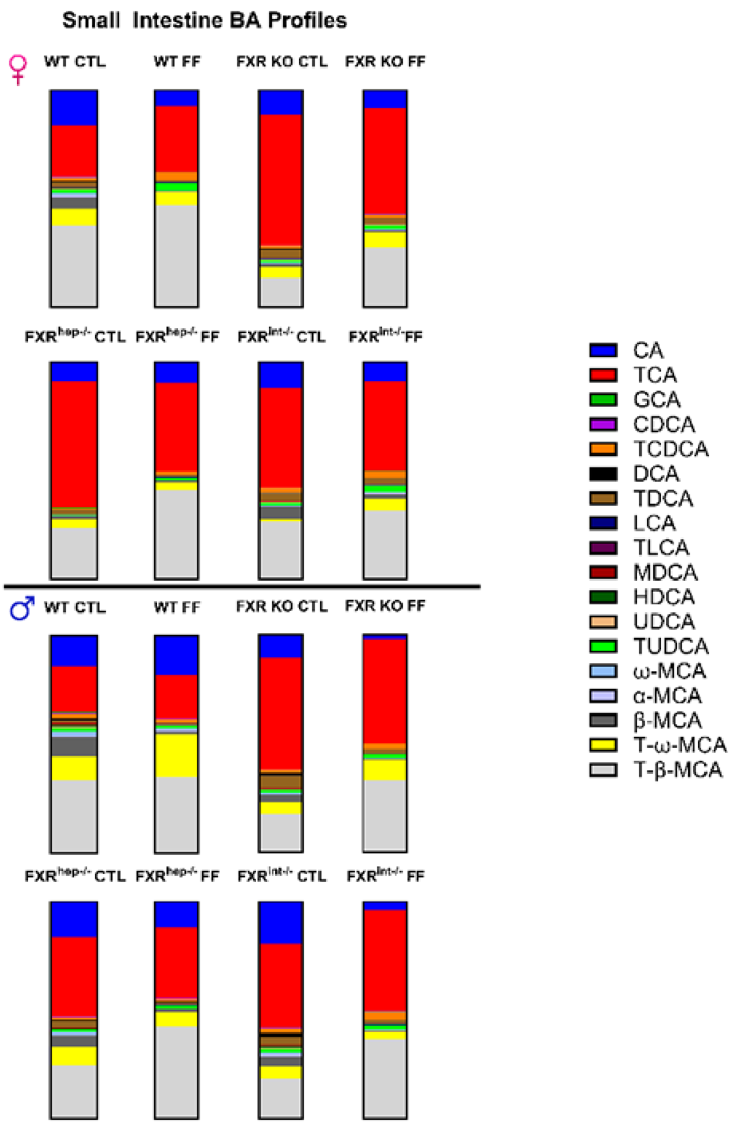

Supplementary Figure 6. Small Intestine BA Profiles. Female (top) and male (bottom) SI individual BA species relative abundance.

A. Individual BAs in Serum

Individual BAs (nM)	FWT CTL	FWT FF	FFXRKO CTL	FFXRKO FF	FFXR$^{hep-/-}$ CTL	FFXR$^{hep-/-}$ FF	FFXR$^{int-/-}$ CTL	FFXR$^{int-/-}$-FF	MWT CTL	MWT FF	MFXRKO CTL	MFXRKO FF	MFXR$^{hep-/-}$ CTL	MFXR$^{hep-/-}$ FF	MFXR$^{int-/-}$ CTL	MFXR$^{int-/-}$-FF
CA	1050.46±418.74	414.70±351.26	2209.18±2672.76	1158.32±1290.62	1408.89±635.92	1354.75±777.95	1704.23±1860.26	848.69±505.03	586.59±290.14	858.91±237.02	644.61±614.92	211.26±162.69	1472.99±750.53	822.71±582.60	858.04±292.56	282.00±272.27
TCA	426.47±332.74	1492.17±1985.95	4250.94±3327.06	4649.99±2716.60	4709.47±5265.82	16854.36±17530.43	1048.02±1137.81	888.12±553.08	253.21±128.78	3832.93±2122.87	576.25±482.14	1185.53±700.57	755.43±546.84	3008.34±2299.80	174.09±53.51	2689.43±3483.46
GCA	0.00±0.00	0.00±0.00	0.00±0.00	0.00±0.00	0.00±0.00	0.00±0.00	0.00±0.00	0.00±0.00	0.00±0.00	0.00±0.00	0.00±0.00	0.00±0.00	0.00±0.00	0.00±0.00	0.00±0.00	0.00±0.00
CDCA	93.25±51.37	63.75±22.00	175.96±156.86	48.05±58.80	73.34±31.34	58.95±70.97	127.96±53.21	91.96±27.16	129.55±68.55	96.59±67.37	100.02±124.58	52.39±41.43	76.23±27.41	68.63±60.51	119.87±81.35	54.51±38.70
TCDCA	69.58±66.87	153.19±152.28	92.47±50.80	168.98±83.39	39.00±25.38	803.55±395.58	41.91±11.75	86.03±10.69	36.63±19.84	246.09±135.32	67.80±74.10	113.92±96.93	58.83±27.56	131.89±55.30	28.22±7.21	181.40±174.65
DCA	801.32±215.76	266.08±193.23	2807.37±2592.90	1052.37±510.25	661.35±312.69	696.55±264.52	875.51±317.09	686.56±395.87	408.93±146.61	321.98±88.76	622.97±550.06	153.29±19.69	422.51±157.39	286.31±93.51	596.73±409.62	269.43±161.07
TDCA	148.16±62.59	165.62±184.05	1223.47±976.92	716.64±416.68	438.38±564.09	861.66±411.20	211.58±82.75	350.21±219.52	82.84±33.73	201.53±103.83	264.07±270.20	142.31±70.39	203.44±158.63	237.63±168.30	136.09±69.90	270.63±252.75
LCA	356.58±216.77	131.77±26.79	247.73±58.13	180.16±37.08	185.82±13.44	179.29±21.92	167.71±40.11	85.49±76.12	464.47±400.01	104.35±70.75	201.05±58.89	77.26±50.50	136.87±27.52	140.54±53.02	128.97±56.17	177.53±23.79
TLCA	17.98±12.13	17.12±8.05	16.98±3.76	19.60±8.17	12.97±6.25	17.37±4.91	8.22±7.38	7.97±3.18	5.52±4.19	9.86±2.97	7.44±3.57	4.54±3.57	6.28±4.98	9.07±6.59	6.66±6.27	1.62±3.25
MDCA	239.47±143.96	253.90±111.36	281.41±96.46	311.89±66.58	195.45±109.81	351.55±163.35	296.24±26.69	194.74±56.34	669.40±480.62	684.62±562.15	380.37±45.55	290.54±156.00	264.44±139.07	171.84±105.71	188.11±138.18	176.88±50.14
HDCA	355.74±217.28	576.71±160.85	434.50±403.93	296.04±169.77	109.59±131.16	57.18±29.19	63.76±57.14	59.97±64.71	456.52±346.17	471.86±428.30	139.77±136.26	139.84±63.79	98.61±105.89	102.29±111.64	207.18±120.63	65.52±19.60
UDCA	251.18±130.62	221.17±164.30	361.24±351.69	248.05±76.32	78.29±67.65	122.42±61.95	140.95±17.33	170.62±116.93	535.43±398.09	658.69±695.51	202.11±69.91	97.63±31.27	101.22±52.91	67.02±54.10	110.29±33.45	55.83±11.49
TUDCA	50.72±34.34	91.16±55.56	82.74±49.66	134.97±51.92	20.27±4.30	293.59±151.07	35.19±4.74	100.72±26.27	31.41±6.76	95.86±43.08	25.08±25.16	51.61±20.37	33.20±26.96	73.58±34.56	25.79±9.68	96.41±78.27
ω-MCA	1278.16±882.88	185.74±169.31	1525.09±1670.22	925.55±775.47	392.26±132.05	309.18±77.74	282.97±326.07	603.55±343.30	630.11±283.67	548.86±96.27	138.35±136.34	86.29±67.94	526.35±579.26	129.30±100.75	271.27±168.05	81.38±50.79
α-MCA	150.21±128.70	61.34±13.05	99.85±92.65	101.03±45.81	23.04±22.32	52.11±27.14	86.63±41.56	146.89±83.34	46.30±8.88	36.52±10.78	26.89±46.57	33.48±17.61	91.82±31.29	37.03±27.37	60.47±40.14	54.09±44.64
β-MCA	1668.37±1159.12	289.19±90.26	1241.02±1666.25	1010.32±738.45	600.78±107.28	1147.56±518.55	855.70±700.92	763.44±402.09	817.27±371.28	318.26±57.12	326.69±235.85	365.76±106.90	850.29±446.63	606.37±218.90	434.07±281.28	325.94±147.93
T-ω-MCA	233.50±149.80	263.13±311.77	351.67±240.46	757.85±506.69	240.58±119.44	1334.66±617.11	68.99±48.40	253.85±169.43	125.12±69.92	1447.37±746.53	63.39±66.96	183.03±112.91	198.53±188.43	305.86±176.50	60.10±11.59	201.49±194.34
T-α-MCA	132.76±91.22	307.17±292.73	175.53±155.60	515.38±282.68	146.38±54.65	751.59±1043.58	135.46±74.26	210.23±88.83	71.15±30.56	643.18±296.28	63.39±76.11	138.98±103.61	79.81±54.50	61.84±86.74	39.11±4.92	195.78±358.16
T-β-MCA	308.07±297.39	720.00±641.14	455.76±310.82	1254.59±789.13	465.39±339.97	8272.91±4947.06	223.60±171.85	298.28±115.22	215.24±111.05	1601.47±899.73	109.31±100.39	393.74±182.70	240.43±165.37	1358.67±749.80	81.25±30.11	1064.84±1318.49

B. Individual BAs in Liver

Individual BAs (nmo/lg)	FWT CTL	FWT FF	FFXRKO CTL	FFXRKO FF	FFXR$^{hep-/-}$ CTL	FFXR$^{hep-/-}$ FF	FFXR$^{int-/-}$ CTL	FFXR$^{int-/-}$-FF	MWT CTL	MWT FF	MFXRKO CTL	MFXRKO FF	MFXR$^{hep-/-}$ CTL	MFXR$^{hep-/-}$ FF	MFXR$^{int-/-}$ CTL	MFXR$^{int-/-}$-FF
CA	1.93±0.82	0.78±0.46	3.99±2.44	2.06±1.67	2.68±1.08	2.05±0.85	2.21±0.99	1.13±0.26	1.90±0.61	0.86±0.23	1.81±0.84	1.56±0.47	2.70±1.03	3.18±2.89	2.07±0.21	0.45±0.05
TCA	71.45±22.11	50.21±32.31	312.58±131.78	126.05±31.44	182.23±14.61	421.08±93.22	136.93±39.85	95.60±52.97	64.67±22.44	45.37±12.10	69.89±42.18	61.72±16.93	118.09±65.00	173.12±41.42	66.00±11.01	38.19±9.28
GCA	0.47±0.11	0.30±0.06	0.54±0.23	0.57±0.26	0.68±0.10	1.52±0.26	0.42±0.15	0.41±0.23	0.51±0.22	0.21±0.09	0.35±0.25	0.55±0.30	0.94±0.33	1.06±0.16	0.26±0.15	0.25±0.02
CDCA	0.09±0.06	0.32±0.29	0.39±0.09	0.37±0.21	0.11±0.07	0.39±0.03	0.23±0.19	0.18±0.08	0.31±0.15	0.31±0.18	0.37±0.30	0.66±0.18	0.18±0.09	0.40±0.21	0.10±0.05	0.07±0.05
TCDCA	2.95±1.18	6.75±3.40	5.36±2.21	6.82±2.04	2.66±0.82	8.84±2.46	8.41±5.04	7.22±3.89	3.14±0.84	3.27±0.56	3.77±1.36	4.80±1.23	4.69±1.14	6.22±1.31	3.50±0.32	4.01±1.06
DCA	0.28±0.16	0.13±0.09	0.74±0.62	0.29±0.05	0.29±0.12	0.21±0.09	0.15±0.08	0.20±0.18	0.50±0.09	0.45±0.06	0.63±0.18	0.53±0.08	0.59±0.15	0.56±0.01	0.61±0.13	0.44±0.06
TDCA	11.94±2.77	5.87±2.00	43.22±22.17	17.66±4.27	13.51±5.94	12.60±3.96	23.55±13.64	13.71±6.28	9.26±1.16	2.97±1.31	15.33±7.51	8.12±2.50	15.32±9.41	12.62±1.17	16.99±7.71	4.13±1.27
LCA	0.23±0.25	0.45±0.35	0.10±0.13	0.00±0.00	0.46±0.39	1.01±0.83	1.30±0.36	0.42±0.43	1.26±0.53	1.06±0.57	2.01±0.66	1.03±0.68	1.23±0.69	1.45±0.42	1.39±0.31	0.83±0.61
TLCA	0.35±0.02	0.27±0.03	0.29±0.02	0.26±0.05	0.12±0.03	0.26±0.09	0.53±0.29	0.27±0.10	0.37±0.06	0.19±0.04	0.31±0.14	0.43±0.14	0.33±0.08	0.42±0.12	0.41±0.11	0.21±0.05
MDCA	0.74±0.19	0.69±0.24	1.00±0.24	0.85±0.41	0.66±0.15	0.71±0.16	0.73±0.34	0.63±0.32	1.60±0.34	0.58±0.15	1.12±0.12	0.95±0.38	1.50±0.60	0.54±0.14	0.76±0.12	0.57±0.31
UDCA	0.34±0.09	0.24±0.15	0.58±0.43	0.47±0.13	0.13±0.17	0.62±0.35	0.30±0.12	0.24±0.16	0.54±0.06	0.14±0.14	0.58±0.43	0.58±0.22	0.48±0.22	0.68±0.13	0.15±0.15	0.21±0.20
TUDCA	5.14±2.20	3.98±1.49	8.31±1.64	5.80±2.34	2.33±1.01	10.95±5.30	5.18±2.53	5.47±1.25	5.78±1.20	1.34±0.21	2.94±1.89	2.97±0.35	5.39±1.92	5.66±1.66	3.28±0.33	1.93±0.66
ω-MCA	1.51±1.12	0.41±0.19	1.92±1.09	1.44±0.71	0.77±0.29	1.00±0.27	0.31±0.23	0.52±0.23	1.85±0.93	1.20±0.29	0.65±0.31	1.70±0.69	2.60±1.96	1.42±0.49	0.75±0.14	0.09±0.07
α-MCA	0.35±0.00	0.35±0.00	0.35±0.00	0.35±0.00	0.35±0.00	0.35±0.00	0.35±0.00	0.35±0.00	0.35±0.00	0.35±0.00	0.35±0.00	0.35±0.00	0.35±0.00	0.35±0.00	0.35±0.00	0.35±0.00
β-MCA	10.75±3.44	7.94±1.81	14.78±9.42	14.79±5.03	9.13±2.57	60.01±19.94	5.66±0.92	7.91±5.66	18.98±10.49	6.96±1.36	9.34±5.87	23.24±12.04	32.47±12.75	41.02±4.06	6.81±5.03	5.18±1.40
T-ω-MCA	18.78±6.21	9.26±5.29	27.02±9.60	22.95±12.70	16.58±5.36	26.71±6.64	7.95±4.96	12.20±5.11	23.03±11.37	17.93±2.89	5.94±3.67	11.71±7.20	32.61±22.52	17.68±8.21	9.23±1.90	2.35±0.73
T-α-MCA	0.00±0.00	0.00±0.00	0.00±0.00	0.00±0.00	0.00±0.00	0.00±0.00	0.00±0.00	0.00±0.00	0.00±0.00	0.00±0.00	0.00±0.00	0.00±0.00	0.00±0.00	0.00±0.00	0.00±0.00	0.00±0.00
T-β-MCA	80.49±30.34	44.52±9.41	82.31±21.61	80.83±33.72	74.69±23.73	381.59±137.92	52.41±9.22	58.23±17.59	82.01±39.91	27.27±8.11	22.84±15.03	38.70±19.87	112.46±40.32	115.17±1.91	29.88±14.16	26.51±9.67

C. Individual BAs in Small Intestine

Individual BAs (nmol/g)	FWT CTL	FWT FF	FFXRKO CTL	FFXRKO FF	FFXR$^{hep-/-}$ CTL	FFXR$^{hep-/-}$ FF	FFXR$^{int-/-}$ CTL	FFXR$^{int-/-}$-FF	MWT CTL	MWT FF	MFXRKO CTL	MFXRKO FF	MFXR$^{hep-/-}$ CTL	MFXR$^{hep-/-}$ FF	MFXR$^{int-/-}$ CTL	MFXR$^{int-/-}$-FF
CA	1653.78±514.79	1067.41±1101.29	1209.93±858.95	1059.93±1067.02	1066.65±252.17	1511.01±918.68	1107.83±485.38	1140.94±889.52	087.53±347.0	1883.11±258.78	948.93±788.34	336.70±331.41	1620.82±565.17	1268.31±1234.41	1378.39±516.10	387.32±352.84
TCA	1881.79±282.59	3547.33±1365.91	5124.00±1310.40	4839.62±1531.06	5727.41±1863.39	5144.71±915.01	3447.02±1271.56	4151.70±1108.68	318.31±119.7	1615.29±279.06	3668.61±1580.29	6195.03±1817.21	2934.15±1007.27	2875.83±1391.82	2172.83±1177.59	3735.22±529.54
GCA	16.59±5.29	11.17±3.96	1.82±2.36	8.07±4.59	19.28±6.76	9.61±1.18	15.77±2.81	11.82±3.36	11.97±4.63	5.61±1.11	10.97±1.68	8.22±1.55	15.80±3.20	6.38±1.35	11.33±3.18	7.84±4.10
CDCA	38.17±12.12	35.41±24.94	24.22±15.93	15.56±7.09	14.26±2.91	23.13±12.92	29.82±13.94	38.51±16.97	41.84±14.54	44.55±18.45	29.50±14.00	9.75±7.73	31.69±10.41	23.12±17.84	39.81±9.14	12.82±3.13
TCDCA	113.86±16.56	394.24±148.33	93.45±53.16	164.98±86.94	52.56±12.90	202.05±66.66	161.20±123.83	287.83±175.39	142.53±43.87	133.25±27.66	108.76±55.46	338.79±127.48	90.09±25.89	125.85±59.92	120.30±62.74	307.27±65.19
DCA	85.97±41.08	19.39±21.24	85.69±77.79	30.97±34.62	23.13±9.58	16.74±8.96	53.23±32.10	40.80±43.72	72.97±8.61	23.03±6.97	106.45±96.19	11.48±9.17	94.31±32.40	18.78±16.56	145.88±63.78	6.73±4.90
TDCA	151.69±42.84	166.71±50.77	361.93±133.46	293.02±88.61	193.56±88.31	119.46±33.37	255.62±273.20	324.80±102.35	67.93±29.16	42.72±7.42	417.64±495.85	280.79±100.23	230.07±115.60	132.70±112.33	204.74±82.96	182.67±24.21
LCA	25.65±18.07	40.91±6.02	39.10±1.88	37.37±2.38	24.00±16.04	36.73±5.01	28.63±4.60	23.47±16.04	25.71±17.36	26.24±18.55	28.89±19.64	30.19±2.61	22.40±15.12	27.23±2.71	27.26±6.66	22.53±15.09
TLCA	3.90±0.84	3.66±0.35	2.16±1.45	3.46±0.56	2.02±0.74	1.62±0.36	4.58±2.54	3.68±1.15	2.48±0.89	2.20±1.07	2.49±1.10	2.13±1.69	1.83±0.38	1.87±0.47	2.44±1.50	3.35±0.91
MDCA	43.23±16.71	18.75±15.67	21.47±19.92	12.81±15.28	18.90±7.49	10.35±7.98	18.14±13.22	18.02±2.52	56.94±25.55	24.35±13.89	26.53±22.41	15.23±10.41	27.02±10.64	15.95±10.85	49.58±23.74	13.06±9.61
HDCA	48.76±25.03	12.92±9.50	18.27±10.42	15.87±10.76	14.40±6.64	8.98±2.86	13.96±1.52	12.80±8.30	85.45±24.78	18.84±3.70	29.68±23.97	18.71±12.51	55.69±29.61	11.06±9.24	55.64±19.76	6.33±2.11
UDCA	40.22±16.04	17.39±14.75	18.83±12.94	15.98±4.07	9.30±6.33	13.13±6.92	36.19±4.09	30.45±22.35	58.22±15.42	16.68±5.97	26.82±12.30	13.36±5.80	37.29±21.12	12.10±5.57	40.36±11.18	7.97±5.70
TUDCA	150.56±23.37	396.98±184.85	88.31±38.33	185.55±67.62	47.05±17.12	139.43±57.05	97.97±76.39	274.10±122.91	105.46±24.94	102.13±20.63	88.40±49.78	218.26±61.73	84.28±14.66	108.73±8.25	100.94±48.66	186.84±63.14
ω-MCA	105.21±27.67	30.16±31.16	64.59±48.56	58.57±65.44	36.18±23.43	21.75±7.54	30.82±12.18	59.78±49.04	148.11±68.67	112.66±22.23	61.89±60.19	20.32±19.35	121.91±106.65	22.29±21.99	68.90±30.53	7.43±7.21
α-MCA	83.17±27.82	31.85±18.50	12.45±8.46	31.90±28.06	9.39±3.07	10.64±7.35	33.28±23.68	109.27±78.32	33.23±4.76	49.24±17.51	16.40±11.42	15.90±14.24	49.76±34.29	23.57±17.93	61.50±34.59	23.21±18.83
β-MCA	541.22±186.95	77.18±53.03	185.82±169.38	129.06±112.36	197.40±108.97	151.98±79.59	569.43±578.31	229.71±189.75	721.37±318.79	140.31±42.07	330.47±327.73	74.14±60.33	543.61±222.54	154.84±161.94	332.68±269.71	52.29±35.34
T-ω-MCA	631.72±85.54	700.47±256.46	427.29±172.53	689.37±207.58	400.33±115.55	466.45±86.24	61.79±107.03	581.82±125.12	686.21±112.52	1601.10±193.01	374.51±106.02	1233.78±570.33	666.15±221.17	557.81±236.59	319.52±47.87	266.96±182.24
T-α-MCA	0.00±0.00	0.00±0.00	0.00±0.00	0.00±0.00	0.00±0.00	0.00±0.00	0.00±0.00	0.00±0.00	0.00±0.00	0.00±0.00	88.21±65.96	0.00±0.00	0.00±0.00	0.00±0.00	122.97±115.21	0.00±0.00
T-β-MCA	2950.36±424.91	5421.00±1606.69	1112.09±371.24	2688.23±511.31	2257.98±1126.75	5111.03±1021.63	1958.06±974.42	3137.24±895.53	037.56±250.0	2831.66±333.22	1215.21±498.23	4188.12±939.15	1937.99±245.69	3672.35±727.23	1015.42±730.41	2905.98±609.30

Supplementary Table 3. Individual BA Species in Serum, Liver, and Small Intestine. A.) Individual BAs in Serum, B.) Individual BAs in Liver, C.) Individual BAs in SI. Data in table are presented as mean ± SD (n=3-4).

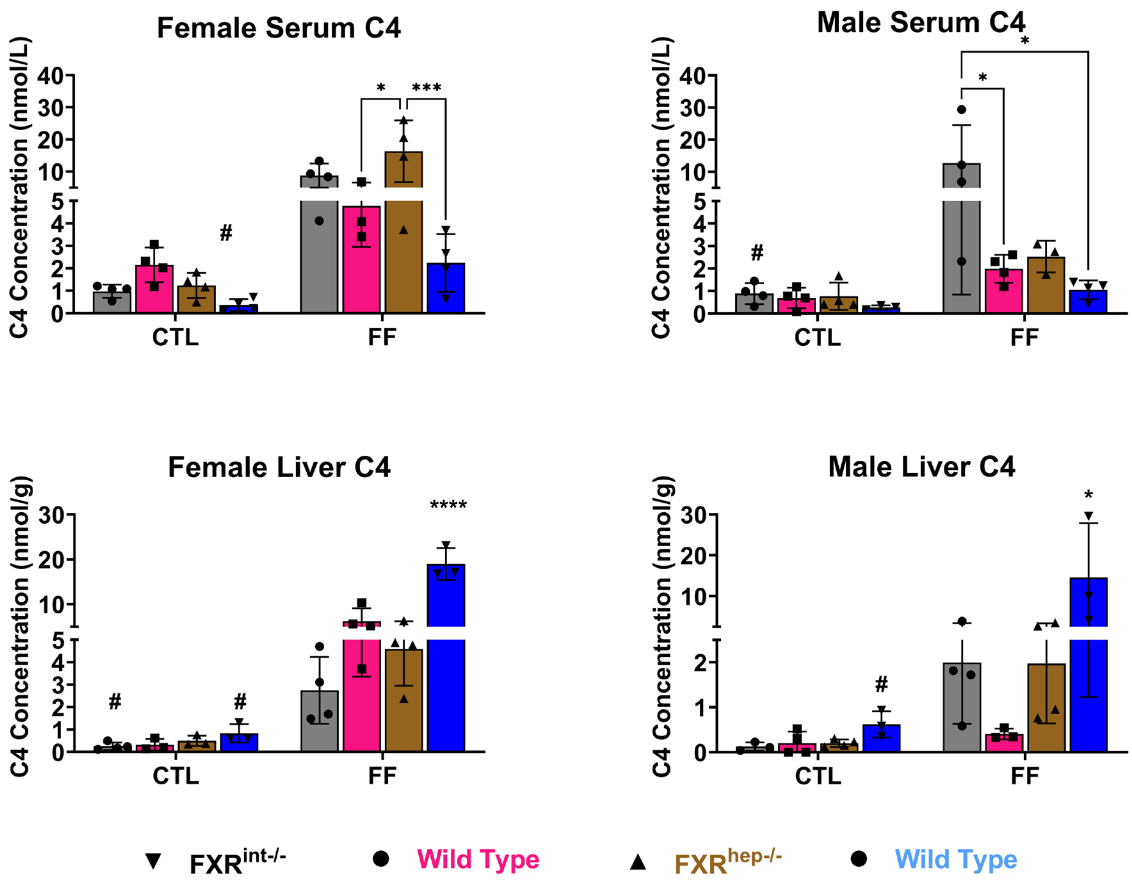

Supplementary Figure 7. C4 Levels in Serum and Liver. LC-MS/MS measured BA intermediate C4 levels. Data represented as mean ± SD (n = 4-15). Two-way ANOVA. $*p < 0.05$, $**p<0.01$, $***p<0.001$, $****p<0.0001$.

	4M MASH Study		
	Average LOQ Values		
	Serum (nM)	Liver (nmol/g)	Intestine (nmol/g)
CA	98.20	0.25	11.05
TCA	48.61	0.10	1283.03
GCA	NF	0.22	4.63
CDCA	255.54	1.02	25.55
TCDCA	60.20	0.12	20.07
DCA	127.77	0.26	12.78
TDCA	60.20	0.10	19.57
LCA	798.87	2.13	53.26
TLCA	NF	0.15	4.66
MDCA	511.20	1.79	76.68
HDCA	511.12	NF	NF
UDCA	255.55	1.02	38.33
TUDCA	40.13	0.20	24.08
ω-MCA	245.54	0.74	24.55
α-MCA	122.75	0.49	18.41
β-MCA	122.77	0.49	24.55
T-ω-MCA	87.50	0.19	163.15
T-α-MCA	58.33	NF	NF
T-β-MCA	97.19	0.19	369.34
C4	1304.33	1.30	NF

Supplementary Table 4. BA Limit of Quantification (LOQ) Values. The LOQ values were determined for each individual BA specie in the serum, liver, and intestine. The LOQs were concentrations with signal-to-noise ratio (S/N) ≥ 3. *NF = not found

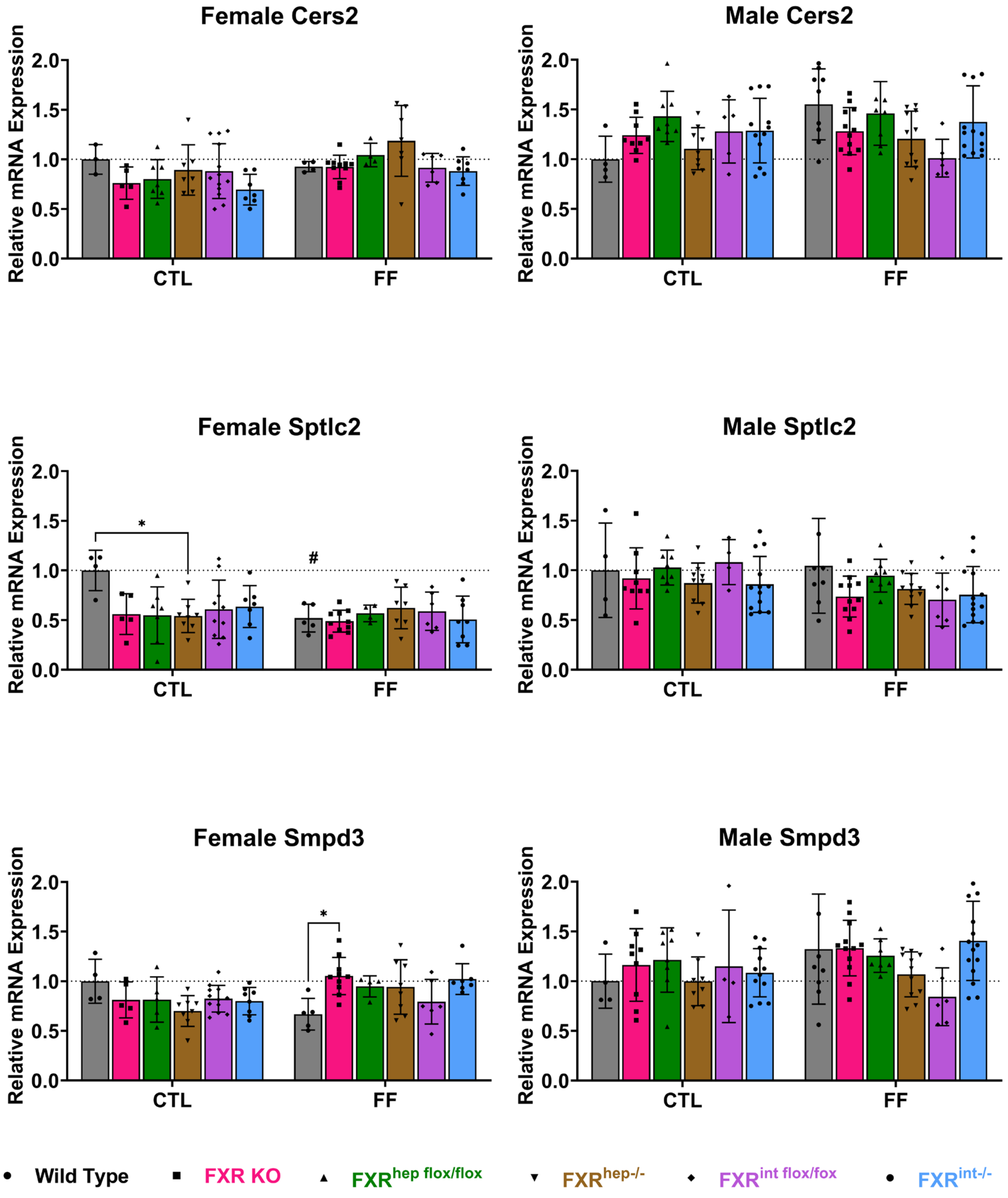

Supplementary Figure 8. Ceramide Synthesis mRNA Expression in the Ileum. Female (left) and male (right) ceramide synthesis gene mRNA expression: Cers2, Sptlc2, Smpd3. Data represented as mean ± SD (n = 4-20). Two-way ANOVA. *p < 0.05, **p<0.01, ***p<0.001, ****p<0.0001. . * Represents significant difference between genotypes and within diet; # Represents significant difference within genotype and across diet.

A. Female Liver Control Diet (FXR$^{hep-/-}$ vs FXR$^{int-/-}$)

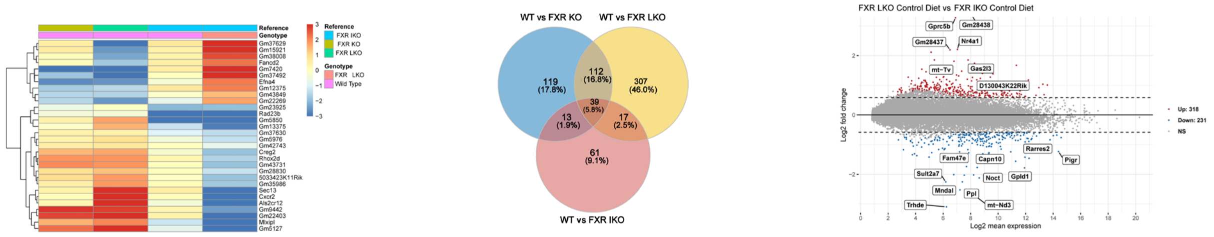

B. Female Liver Fast Food Diet (FXR$^{hep-/-}$ vs FXR$^{int-/-}$)

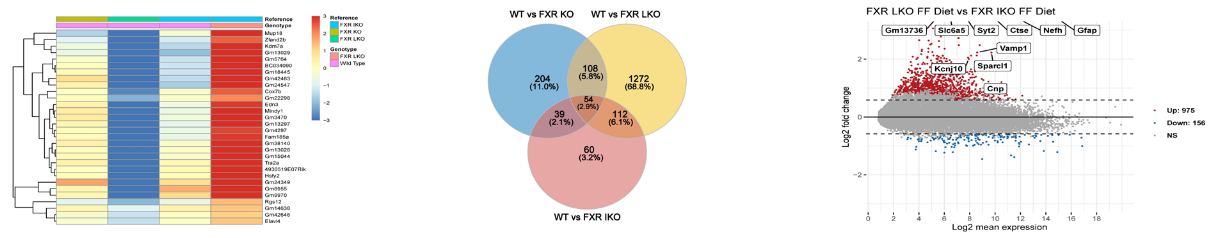

C. Male Liver Control Diet (FXR$^{hep-/-}$ vs FXR$^{int-/-}$)

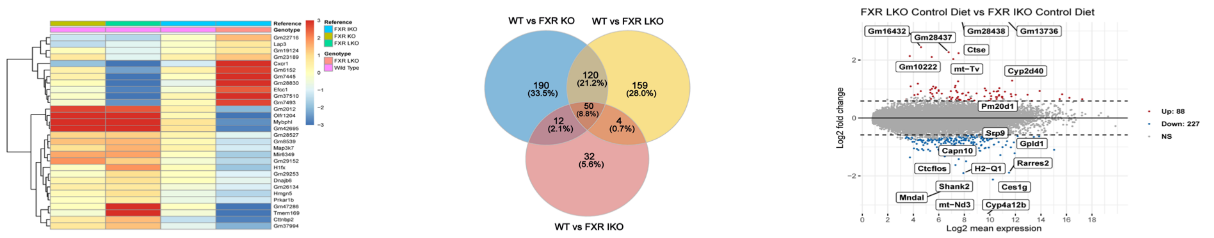

D. Male Liver Fast Food Diet (FXR$^{hep-/-}$ vs FXR$^{int-/-}$)

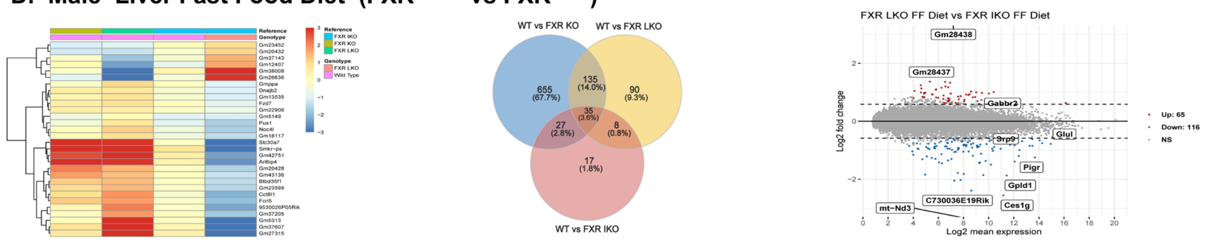

Supplementary Figure 9. RNAseq Analysis (FXR$^{hep-/-}$ vs FXR$^{int-/-}$) in the Liver. A.) Heat map (left), Venn diagram (center), MA plot (right) of liver samples from female FXR$^{hep-/-}$ vs FXR$^{int-/-}$ mice fed control diet; B.) Heat map (left), Venn diagram (center), MA plot (right) of liver samples from female FXR$^{hep-/-}$ vs FXR$^{int-/-}$ mice fed FF diet; C.) Heat map (left), Venn diagram (center), MA plot (right) of liver samples from male FXR$^{hep-/-}$ vs FXR$^{int-/-}$ mice fed control diet; D.) Heat map (left), Venn diagram (center), MA plot (right) of liver samples from male FXR$^{hep-/-}$ vs FXR$^{int-/-}$ mice fed FF diet. (n = 3 per group)

A. Female Ileum Control Diet (FXR$^{hep-/-}$ vs FXR$^{int-/-}$)

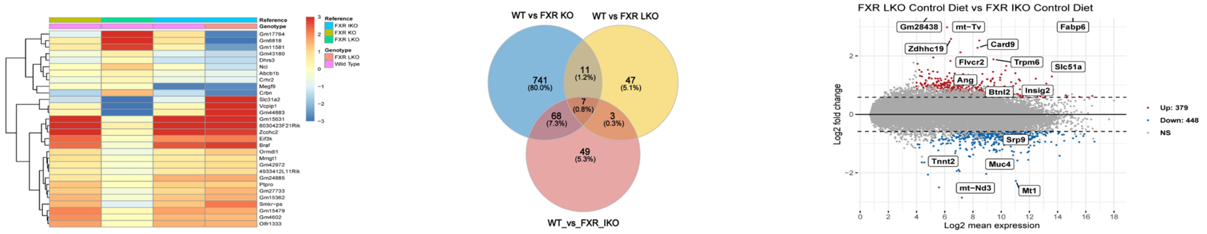

B. Female Ileum Fast Food Diet (FXR$^{hep-/-}$ vs FXR$^{int-/-}$)

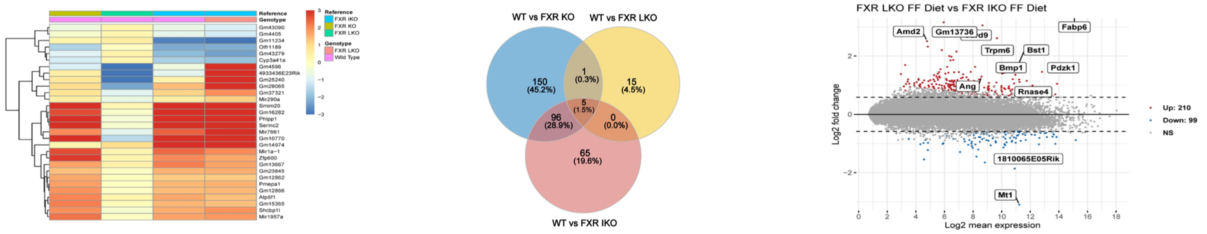

C. Male Ileum Control Diet (FXR$^{hep-/-}$ vs FXR$^{int-/-}$)

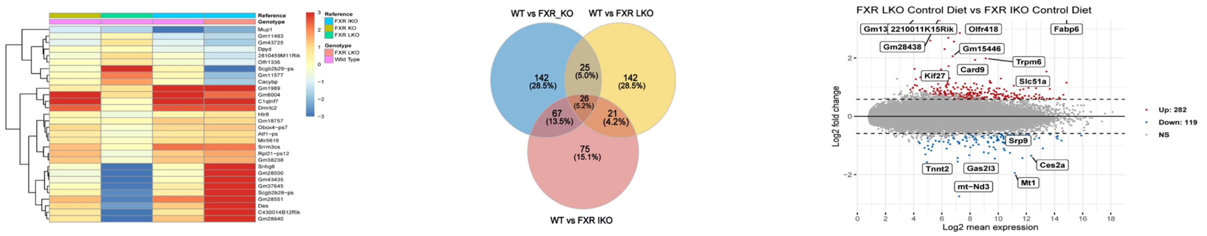

D. Male Ileum Fast Food Diet (FXR$^{hep-/-}$ vs FXR$^{int-/-}$)

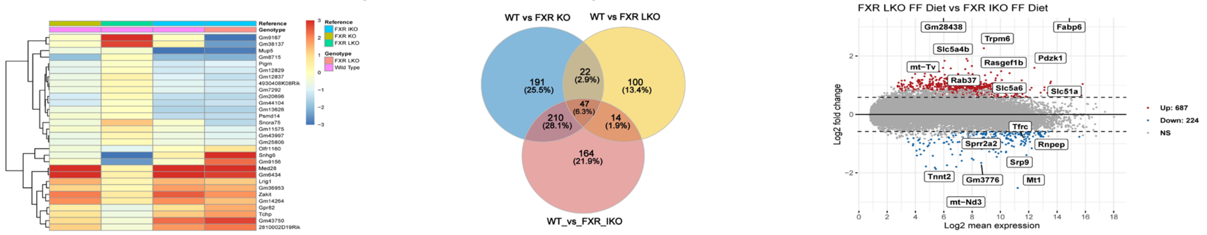

Supplementary Figure 10. RNAseq Analysis (FXR$^{hep-/-}$ vs FXR$^{int-/-}$) in the Ileum. A.) Heat map (left), Venn diagram (center), MA plot (right) of ileum samples from female FXR$^{hep-/-}$ vs FXR$^{int-/-}$ mice fed control diet; B.) Heat map (left), Venn diagram (center), MA plot (right) of ileum samples from female FXR$^{hep-/-}$ vs FXR$^{int-/-}$ mice fed FF diet; C.) Heat map (left), Venn diagram (center), MA plot (right) of ileum samples from male FXR$^{hep-/-}$ vs FXR$^{int-/-}$ mice fed control diet; D.) Heat map (left), Venn diagram (center), MA plot (right) of ileum samples from male FXR$^{hep-/-}$ vs FXR$^{int-/-}$ mice fed FF diet. (n = 3 per group)
